# Identification of core aberrantly expressed microRNAs in serous ovarian carcinoma

**DOI:** 10.18632/oncotarget.24942

**Published:** 2018-04-17

**Authors:** Steven F. Chen, Zheng Liu, Shyambabu Chaurasiya, Thanh H. Dellinger, Jianming Lu, Xiwei Wu, Hanjun Qin, Jinhui Wang, Yuman Fong, Yate-Ching Yuan

**Affiliations:** ^1^ Bioinformatics Core, Beckman Research Institute, City of Hope, Duarte, California 91010, USA; ^2^ Department of Surgery, City of Hope National Medical Center, Duarte, California 91010, USA; ^3^ Integrative Genomics Core, Beckman Research Institute, City of Hope, Duarte, California 91010, USA

**Keywords:** microRNA, serous ovarian carcinoma, omentum, biomarker, small RNA sequencing

## Abstract

MicroRNAs (miRNAs) have recently demonstrated great potential and enormous promise in the diagnosis, prognosis and therapy of various types of cancer. In this study, we performed a comprehensive miRNA expression analysis in the omental metastasis of serous ovarian carcinoma (SOC) using small RNA sequencing. Two hundred and fifty-one aberrantly expressed miRNAs were identified, which clearly separated malignant omentum from normal omentum. Furthermore, miRNA profiles in primary chemo-sensitive and chemo-resistant/refractory SOC were determined using publicly available data. Comparing miRNA expression profiles in omental metastases and primary chemo-sensitive and chemo-resistant/refractory tumors, a set of 70 miRNAs that were aberrantly expressed in both primary and metastatic SOC has been identified for the first time. These core aberrantly expressed miRNAs may play crucial roles in the tumorigenesis, growth, and metastasis of SOC. Therefore, they can serve as potential diagnostic biomarkers and as therapeutic targets for miRNA-mediated therapy. Kaplan–Meier overall survival analysis using The Cancer Genome Atlas data demonstrated that 10 miRNAs (hsa-miR-135, 150, -340, 625, 1908, 3187, -96, -196b, -449c, and -1275) were associated with survival of patients with SOC, which may serve as potential prognostic biomarkers.

## INTRODUCTION

Ovarian cancer is one of the most common gynecological malignancies worldwide, and the most lethal gynecological malignancy in the Western world. Epithelial ovarian cancer (ovarian carcinoma) is the most common type of ovarian cancer, accounting for 90% of ovarian cancer. Ovarian carcinoma can be divided into 4 major histologic subtypes: serous, endometrioid, clear cell, and mucinous [1]. Serous ovarian carcinoma (SOC) accounts for 75–80% of ovarian carcinoma [2]. It is estimated that the annual number of new ovarian cancer cases in the United States will increase by 37% from 20,921 cases in 2010 to 28,591 cases in 2030 [3]. Despite advances in diagnosis and treatment, the overall cure rate (30%) remains unchanged over the past 40 years [4]. The poor outcome is mainly due to lack of effective approaches to early detection and to treating disseminated or recurrent ovarian cancer. Therefore, there is an urgent and unmet need to develop innovative strategies for early diagnosis and effective therapy.

MicroRNAs (MiRNAs) are small, endogenously expressed, non-coding RNAs of 19–25 nucleotides in length. MiRNAs are transcribed in the nucleus by RNA polymerase II as long primary transcripts called primary miRNAs (pri-miRNAs), which are processed by the RNase III-type endonuclease Drosha into precursor miRNAs (pre-miRNAs). The pre-miRNA is exported to the cytoplasm by exportin 5, and then cleaved by Dicer into a mature miRNA. The mature miRNA assembles into the RNA-induced silencing complex (RISC), through which, miRNAs can regulate their targets, leading to translational inhibition or mRNA degradation [5]. The mode of action depends on the level of complementarity between miRNAs and their targets. Perfect complementarity favors mRNA degradation while imperfect complementarity results in translation inhibition [6]. In the most recent version of miRbase (version 21, June 2017), 2588 mature human (*Homo sapiens*) miRNAs and 1915 mature mouse (*Mus musculus*) miRNAs have been identified. It was estimated that more than 60% of human protein-coding genes are regulated by miRNAs [7]. MiRNAs are known to play important roles in many, if not all, normal biological processes, such as cell growth, apoptosis, and differentiation. Dysregulation of miRNAs are associated with many diseases such as neurodegenerative and metabolic disorders as well as cancer [5]. In cancer, miRNAs can function either as oncogenes (oncomirs) or tumor suppressors. The first evidence of dysregulation of miRNAs in cancer was reported by Calin *et al.* in 2002 [8]. The authors found that miR-15a and miR-16-1 located at chromosome 13q14 are deleted or down-regulated in the majority of B cell chronic lymphocytic leukemias, suggesting these miRNAs might function as tumor suppressors. To date, it is evidenced that miRNAs have global alternations in patterns of expression across multiple malignancies including ovarian cancer. Thirty-four miRNAs were found to be consistently dysregulated in ovarian cancer from several independent studies [9]. In a recent study, 1156 miRNAs were found to be de-regulated in ovarian cancer compared to its concurrent endometriosis [10]. These differentially expressed miRNAs may serve as diagnostic biomarkers for ovarian cancer in association with endometriosis. More recently, 59 known and 20 novel miRNAs were found to be differentially expressed between normal tubal tissue and BRCA1-assoicated high-grade serous ovarian carcinoma [11].

In recent years, miRNAs have shown great potential and enormous promise in the diagnosis, prognosis and therapy of various types of cancer including ovarian cancer [12, 13]. Early diagnosis is critical for the successful treatment of any cancer. Unfortunately, due to the insidious asymptomatic nature of ovarian cancer in its early onset, a robust and minimally invasive method for early detection of ovarian cancer is still lacking. Several recent studies indicate the feasibility of using miRNAs as novel diagnostic and prognostic biomarkers for ovarian cancer. In a study comparing sera from patients with ovarian carcinoma and normal subjects, miR-21, -29a, -92, -93, and -126 were found to be overexpressed whereas miR-99b, -127, and -155 were underexpressed in patients with ovarian carcinoma [14]. Zheng and coworkers found higher level of miR-205 and lower level of let-7f in the plasma of patients with ovarian carcinoma compared with healthy controls. Thus, the levels of miR-205 and lef-7f can serve as diagnostic biomarkers for ovarian carcinoma. In addition, lower levels of let-7f were correlated with poor prognosis [15]. The therapeutic application of miRNAs involves suppression of up-regulated miRNAs (oncomirs) or substitution of down-regulated miRNAs (tumor suppressors) to achieve loss or gain of miRNA function, respectively. Overexpression of miR-182 is known to contribute to the aggressiveness of ovarian cancer. Anti-miR-182 treatment was shown to reduce tumor size, local invasion and distant metastasis [16]. MiR-502d-3p is a tumor suppressor, negatively regulating EphA2. Restoration of miR-502d-3p decreased EphA2 expression levels and suppressed tumor growth. Moreover, dual inhibition of EphA2 using nano-liposomes loaded with miR-502d-3p and EphA2 siRNA showed synergistic therapeutic efficacy [17]. The differences in miRNA expression between normal and cancer cells have been exploited to improve tumor-targeting of oncolytic viruses by incorporating multiple tandem copies of artificial miRNA target sequences into 3’-untranslated regions of essential viral genes [18].

Several studies on miRNA profiling of ovarian cancer have been published so far [2, 10, 11, 19–25]. Most of these studies, however, used microarray based approaches that could detect only a few hundred miRNAs, representing only a small portion of the 2588 current available human miRNAs. In this study, we performed a comprehensive miRNA expression analysis in the omental metastasis of SOC using small RNA sequencing and annotation with the most recent version of miRbase (version 21, June 2017) for the first time. In addition, miRNA profiles in primary chemo-sensitive and chemo-resistant/refractory SOC were analyzed using publicly available data. Comparing miRNA expression profiles in omental metastases and primary chemo-sensitive and chemo-resistant/refractory tumors, a set of core miRNAs that were aberrantly expressed in both primary and metastatic SOC was identified. Kaplan–Meier overall survival analysis using The Cancer Genome Atlas (TCGA) data was performed on the dysregulated miRNAs identified.

## RESULTS

### Distinct miRNA signatures in omental metastases compared with the normal omentum

**Figure 1:**
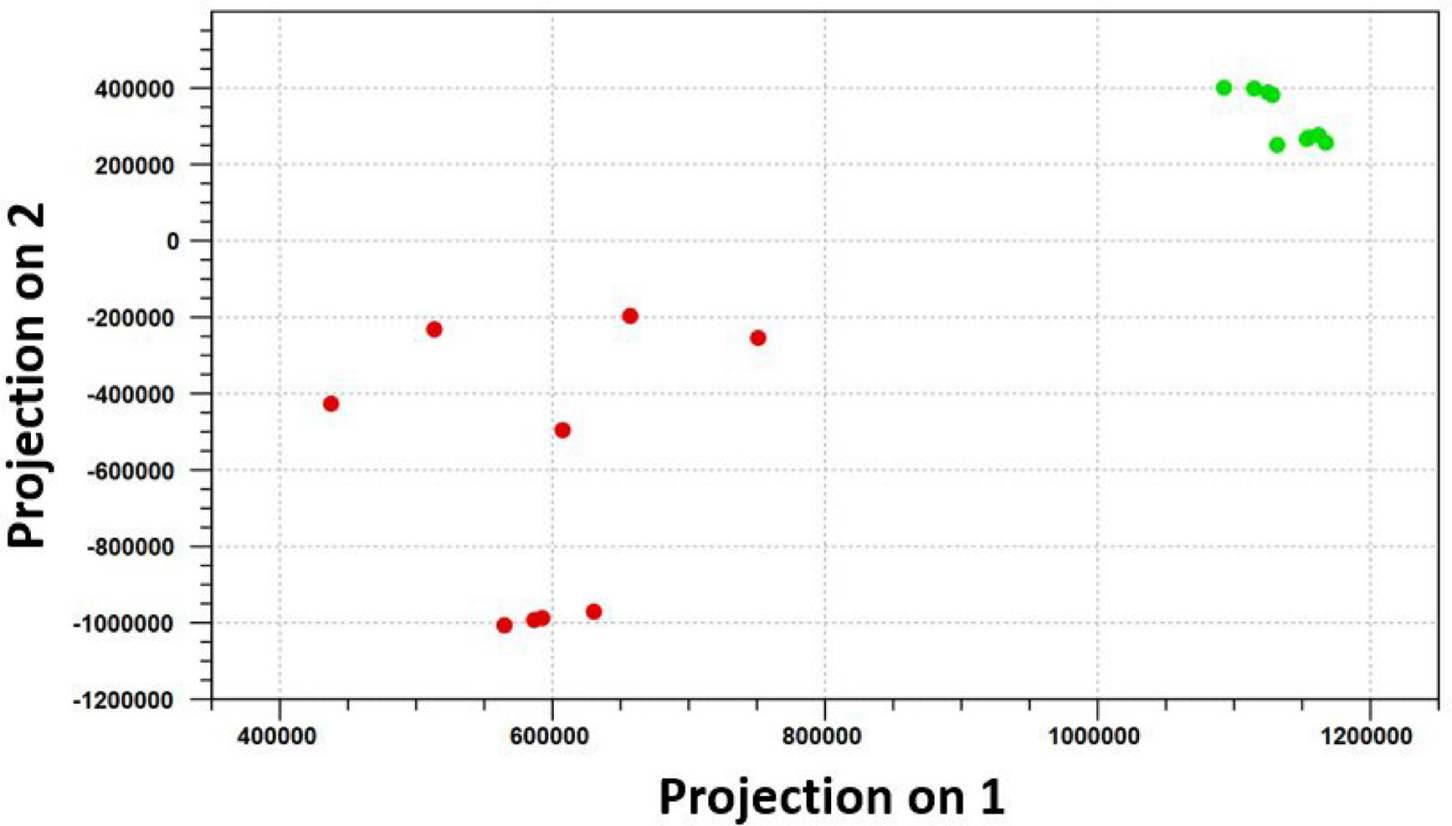
Principal component analysis distinguishes omental metastases perfectly from the normal omentum Principal component analysis of normalized expression values of all annotated miRNAs demonstrated that all normal omental samples closely clustered together, which were clearly separated from all 9 omental metastatic samples that loosely clustered together. The green dots represent the 9 normal omental samples, the red dots represent the 9 omental metastatic samples.

**Figure 2:**
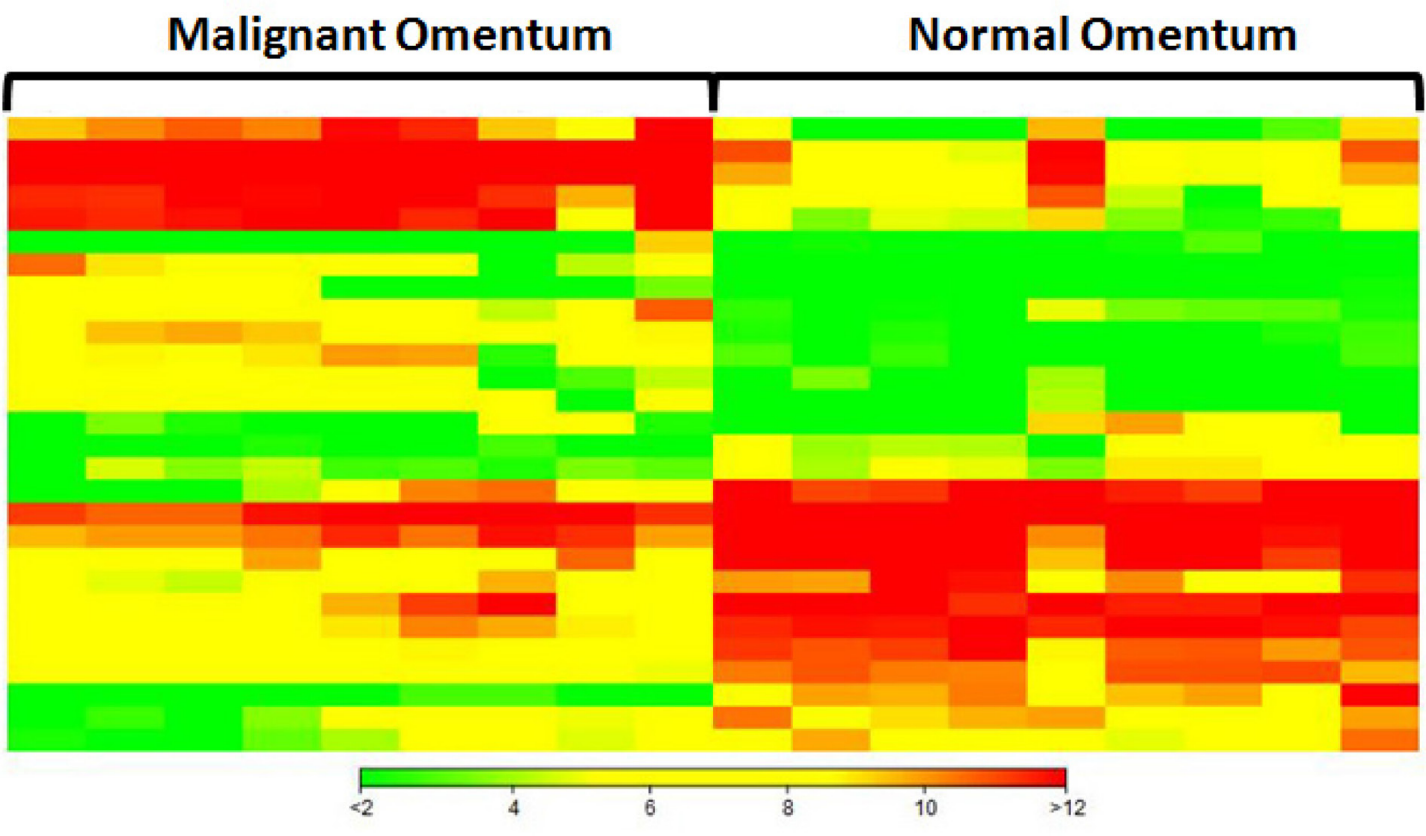
Hierarchical clustering analysis of top 20 up-regulated and top 20 down-regulated miRNAs in omental metastases Each column represents a sample, each row represents a miRNA. Green, yellow, and red represent relative low, medium, and high expression, respectively.

The omentum is a primary site of metastatic spread of ovarian carcinoma. To identify miRNAs that are differentially expressed in omental metastases in comparison with the normal omentum, small RNA sequencing was performed on nine omental metastatic tissues and nine normal omental tissues from a total of seven patients (some patients provided more than one normal or metastatic tissues). Six patients had high-grade SOC, and one patient had anaplastic carcinoma arising in a borderline mucinous cystadenoma of the ovary (Supplementary Table 1). All omental metastatic tissues were obtained from 6 patients with high-grade SOC. Four of them provided both normal and metastatic tissues while the rest of them provided only normal or metastatic tissues. The patient with anaplastic carcinoma provided only normal omental tissues. Principal component analysis of normalized expression values of all annotated miRNAs (Supplementary Table 2) demonstrated that all normal omental samples closely clustered together, which were clearly separated from all 9 omental metastatic samples that loosely clustered together (Figure 1). Hierarchical clustering analysis of top differentially expressed miRNAs indicated distinct miRNA expression patterns between malignant and normal omentum (Figure 2). In total, 251 miRNAs were identified to be significantly differentially expressed (*P* < 0.05, ≥ 2-fold difference in expression level) between malignant and normal omentum, of which, 84 miRNAs were down-regulated, 167 miRNAs were up-regulated in the malignant omentum (Supplementary Table 3). Intriguingly, all members of the miR-200 family (miR-200a, miR-200b, miR-200c, miR-141, and miR-429) were up-regulated. Table 1 lists the top 20 up-regulated and top 20 down-regulated miRNAs (based on fold change) in the malignant omentum.

**Table 1:** Top 20 up-regulated and top 20 down-regulated miRNAs in omental metastases

Up-regulated			Down-regulated		
Name	Fold Change^$^	*P*-value	Name	Fold Change^$^	*P*-value
hsa-miR-449c-5p	1914.70	2.40E-17	hsa-miR-5683-5p	–272.43	6.93E-24
hsa-miR-885-3p	268.22	4.77E-13	hsa-miR-216a-5p	–30.21	2.34E-09
hsa-miR-1269a-3p	170.82	4.66E-09	hsa-miR-144-3p	–23.95	1.16E-05
hsa-miR-449a-5p^#^	166.60	5.17E-15	hsa-miR-1-2-3p^#^	–18.76	8.17E-11
hsa-miR-885-5p	151.76	3.04E-19	hsa-miR-143-3p	–16.77	3.38E-13
hsa-miR-6510-3p	151.48	5.84E-06	hsa-miR-215-5p	–11.81	1.09E-07
hsa-miR-200c-3p^#^	95.20	1.43E-09	hsa-miR-217-5p	–11.63	2.80E-07
hsa-miR-200b-3p^#^	74.15	2.71E-12	hsa-miR-126-5p	–8.83	3.17E-07
hsa-miR-205-5p^*^	71.49	2.52E-05	hsa-miR-451a-5p^#^	–8.53	9.45E-06
hsa-miR-200b-5p	65.77	5.76E-11	hsa-miR-122-5p	–8.45	1.49E-02
hsa-miR-375-3p^#^	50.38	4.45E-09	hsa-miR-511-3p	–8.21	8.89E-08
hsa-miR-891a-5p	46.68	3.15E-04	hsa-miR-144-5p	–7.95	1.41E-04
hsa-miR-187-3p	42.60	3.87E-10	hsa-miR-376a-1-5p	–7.70	2.55E-07
hsa-miR-429-3p	31.00	1.75E-08	hsa-miR-374a-3p	–7.46	5.68E-05
hsa-miR-203b-3p	28.63	3.07E-09	hsa-miR-542-3p	–7.21	1.25E-06
hsa-miR-4697-3p	28.08	2.06E-06	hsa-miR-143-5p	–6.97	9.76E-09
hsa-miR-203a-3p^*^	28.07	1.18E-07	hsa-miR-142-3p	–6.85	2.10E-04
hsa-miR-449b-5p	25.81	5.58E-07	hsa-miR-5701-1-5p	–6.82	1.43E-03
hsa-miR-135b-3p	25.58	1.11E-11	hsa-miR-424-5p	–6.77	6.80E-08
hsa-miR-183-3p	20.71	7.94E-07	hsa-miR-126-3p	–6.65	5.93E-08

^*^concordant with reference 9; ^#^concordant with reference 13; ^$^compared to normal omentum.

### MiRNA profiles in primary chemo-resistant and chemo-sensitive tumors

**Figure 3:**
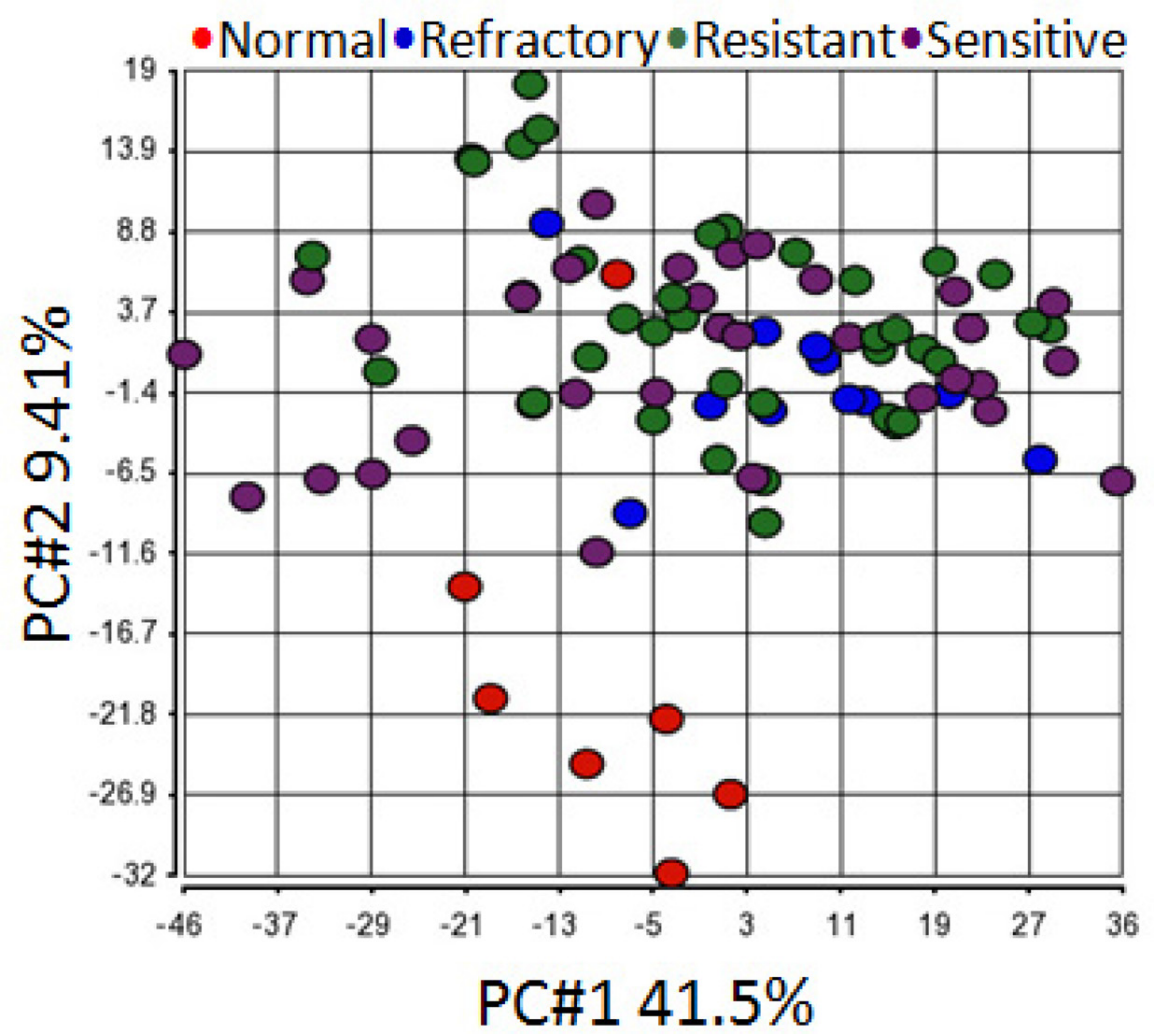
Principal component analysis shows all primary tumor samples clustered together regardless of their chemo-sensitivity Principal component analysis of normalized expression values of all annotated miRNAs demonstrated that all primary tumor samples clustered together irrespective of their sensitivity to chemotherapy whereas 6 normal fallopian tube samples formed a separate cluster. One normal fallopian tube sample that clustered with primary tumor samples was removed without further analysis.

**Figure 4:**
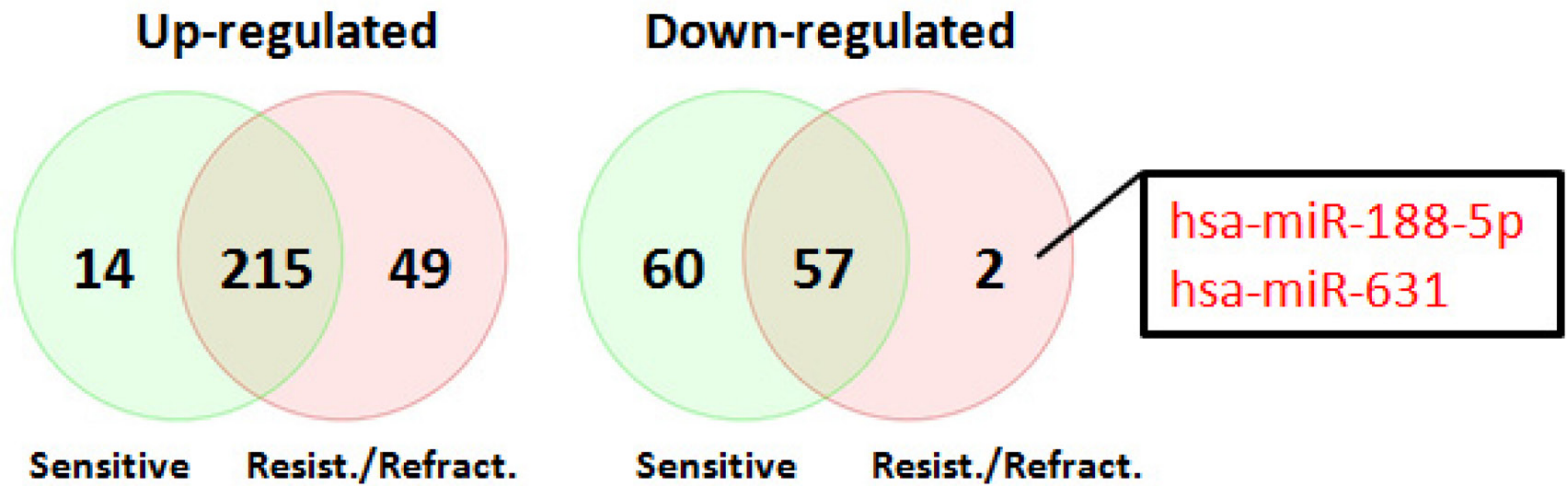
Venn diagrams of differentially expressed miRNAs shared between primary chemo-sensitive tumors and primary resistant/refractory tumors 215 miRNAs including all members of the miR-200 family were up-regulated, and 57 miRNAs were down-regulated in both chemo-sensitive and resistant/refractory tumors. Two miRNAs (hsa-miR-188-5p and hsa-miR-631) were only down-regulated in primary resistant/refractory tumors.

In order to determine miRNA profiles in primary SOC, we searched NCBI Gene Expression Omnibus for public available patient cohorts. Ovarian cancer studies without normal samples and with less than 50 tumor samples were filtered out to ensure statistical significance. We identified the Patch 2015 Nature study to include in our analysis [26]. This study contained 31 primary chemo-sensitive tumors, 37 primary resistant tumors, 12 primary refractory tumors, and 7 normal fallopian tube samples. Principal component analysis of normalized expression values of all annotated miRNAs showed that all primary tumor samples clustered together irrespective of their sensitivity to chemotherapy whereas 6 normal fallopian tube samples formed a separate cluster (Figure 3). One normal fallopian tube sample that clustered with primary tumor samples was removed without further analysis. Compared with normal fallopian tube samples, there were 346 dysregulated miRNAs (229 up-regulated, 117 down-regulated) in primary chemo-sensitive tumors (Supplementary Table 4), 323 dysregulated miRNAs (264 up-regulated, 59 down-regulated) in primary resistant/refractory tumors (Supplementary Table 5). 215 miRNAs including all members of the miR-200 family were up-regulated, and 57 miRNAs were down-regulated in both chemo-sensitive and resistant/refractory tumors (Figure 4). Forty-nine miRNAs were up-regulated, and 2 miRNAs (hsa-miR-188-5p and hsa-miR-631) were down-regulated only in primary resistant/refractory tumors (Supplementary Table 6 and Figure 4). Tables 2 and 3 list top 20 up-regulated and top 20 down-regulated miRNAs (based on fold change) in primary chemo-sensitive tumors and primary resistant/refractory tumors, respectively.

**Table 2:** Top 20 up-regulated and top 20 down-regulated miRNAs in primary chemo-sensitive tumors

Up-regulated			Down-regulated		
Name	Fold Change^*^	*P*-value	Name	Fold Change^*^	*P*-value
hsa-miR-203	257.97	1.61E-16	hsa-miR-767-5p	–19.26	1.58E-03
hsa-miR-143-3p	195.06	1.12E-07	hsa-miR-486-3p	–14.13	9.03E-04
hsa-miR-205-5p	181.37	1.90E-08	hsa-miR-646	–13.95	1.15E-03
hsa-miR-150-5p	152.45	2.66E-08	hsa-miR-938	–13.33	1.70E-03
hsa-miR-451a	138.28	1.07E-08	hsa-miR-562	–10.88	2.80E-03
hsa-miR-199a-3p	117.62	1.63E-09	hsa-miR-3182	–10.80	6.68E-04
hsa-miR-199b-5p	106.57	4.64E-09	hsa-miR-1248	–10.59	1.31E-03
hsa-miR-223-3p	92.67	6.86E-11	hsa-miR-302c-3p	–10.46	2.48E-03
hsa-miR-4286	91.23	4.85E-09	hsa-miR-515-5p	–10.39	2.01E-03
hsa-miR-425-5p	86.45	1.26E-15	hsa-miR-339-3p	–10.24	1.03E-03
hsa-miR-429	78.99	2.60E-14	hsa-miR-586	–10.20	2.84E-03
hsa-miR-513b	67.71	4.36E-04	hsa-miR-548ak	–9.91	2.59E-03
hsa-miR-500a-5p	66.11	2.82E-10	hsa-miR-1288	–9.90	4.27E-03
hsa-miR-1260b	56.98	1.62E-10	hsa-miR-625-5p	–9.73	6.87E-04
hsa-miR-30c-5p	56.52	2.62E-10	hsa-miR-550a-5p	–9.23	5.59E-03
hsa-miR-27a-3p	47.00	5.53E-10	hsa-miR-580	–9.11	2.89E-03
hsa-miR-1915-3p	38.35	6.80E-07	hsa-miR-518a-3p	–9.11	2.27E-03
hsa-miR-575	36.05	1.82E-07	hsa-miR-3136-5p	–9.10	2.65E-03
hsa-miR-345-5p	35.29	2.51E-09	hsa-miR-127-5p	–8.89	6.33E-03
hsa-miR-1246	35.14	1.40E-02	hsa-miR-450b-3p	–8.43	1.42E-03

^*^compared to normal fallopian tube.

**Table 3:** Top 20 up-regulated and top 20 down-regulated miRNAs in primary resistant/refractory tumors

Up-regulated			Down-regulated		
Name	Fold Change^*^	*P*-value	Name	Fold Change^*^	*P*-value
hsa-miR-143-3p	447.34	1.48E-09	hsa-miR-3182	–9.71	9.77E-04
hsa-miR-203	331.38	1.10E-17	hsa-miR-938	–8.75	7.33E-03
hsa-miR-199a-3p	274.37	4.56E-12	hsa-miR-486-3p	–8.61	5.79E-03
hsa-miR-199b-5p	244.71	1.64E-11	hsa-miR-767-5p	–8.60	1.83E-02
hsa-miR-150-5p	173.85	9.56E-09	hsa-miR-3136-5p	–8.54	3.11E-03
hsa-miR-205-5p	159.53	2.70E-08	hsa-miR-548d-5p	–7.74	4.55E-03
hsa-miR-4286	135.54	2.59E-10	hsa-miR-646	–7.63	1.02E-02
hsa-miR-451a	129.19	1.13E-08	hsa-miR-515-5p	–7.31	7.46E-03
hsa-miR-223-3p	116.77	7.86E-12	hsa-miR-550a-5p	–7.25	1.21E-02
hsa-miR-425-5p	92.35	3.75E-16	hsa-miR-576-3p	–6.34	1.76E-02
hsa-miR-500a-5p	85.26	2.61E-11	hsa-miR-518a-3p	–6.04	1.12E-02
hsa-miR-429	84.48	8.15E-15	hsa-miR-1288	–5.61	2.80E-02
hsa-miR-30c-5p	78.16	1.26E-11	hsa-miR-450b-3p	–5.51	9.00E-03
hsa-miR-1246	76.66	2.66E-03	hsa-miR-576-5p	–5.48	3.95E-02
hsa-miR-1260b	76.07	9.97E-12	hsa-miR-520c-3p	–5.27	2.49E-02
hsa-miR-575	61.78	3.24E-09	hsa-miR-548an	–5.18	7.98E-03
hsa-miR-181c-5p	58.45	9.32E-09	hsa-miR-580	–5.18	2.32E-02
hsa-miR-423-5p	54.41	2.14E-12	hsa-miR-3605-5p	–4.99	1.56E-02
hsa-miR-27a-3p	53.54	1.29E-10	hsa-miR-562	–4.99	3.89E-02
hsa-miR-454-3p	42.56	2.36E-07	hsa-miR-499a-3p	–4.97	2.01E-02

^*^compared to normal fallopian tube.

### Dysregulated core miRNAs in both primary and metastatic SOC

**Figure 5:**
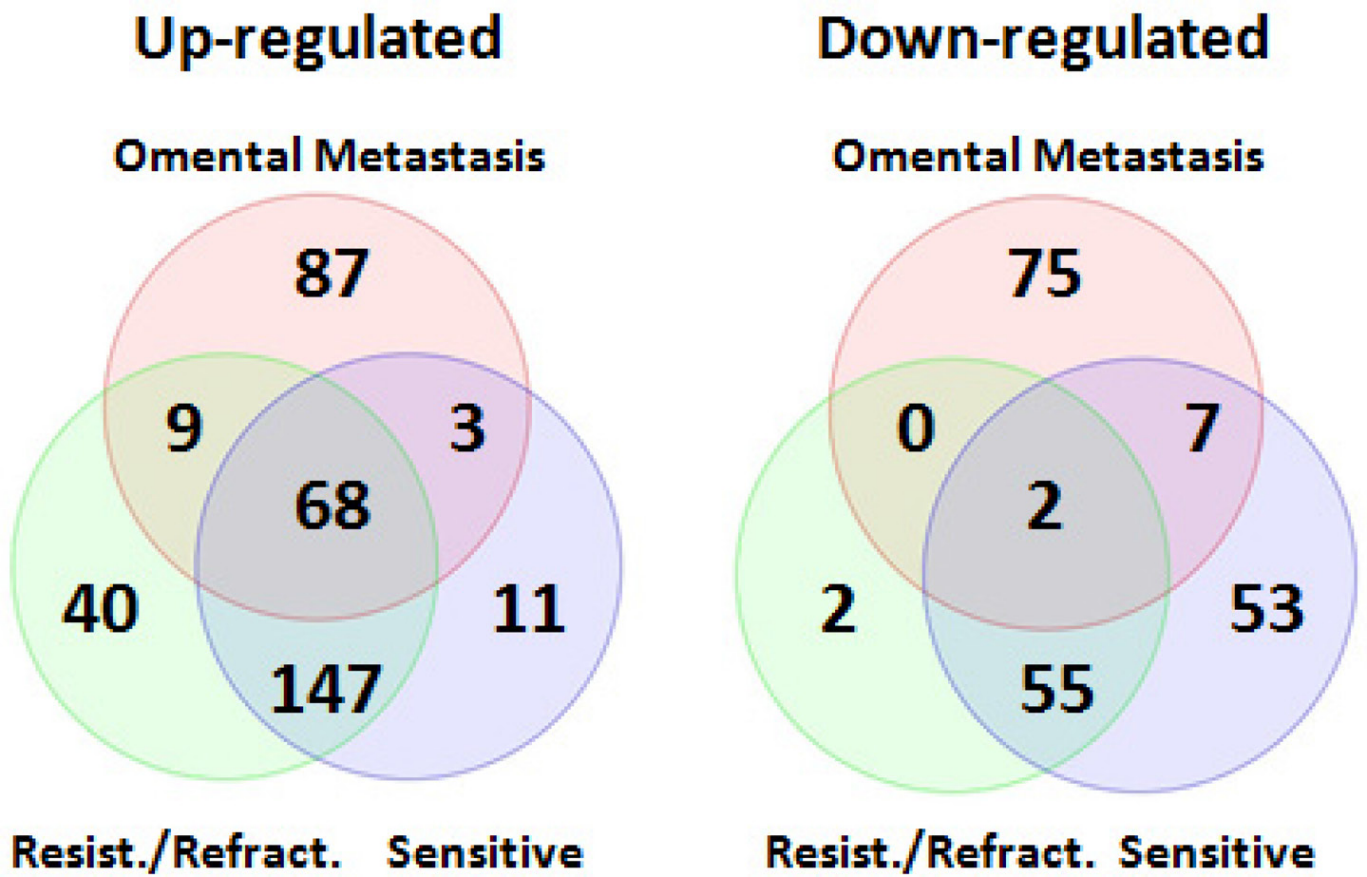
Venn diagrams of differentially expressed miRNAs shared between primary and metastatic serous ovarian carcinoma Comparing miRNA expression profiles in primary SOC (both chemo-sensitive and resistant/refractory) and omental metastases, 68 miRNAs including all members of the miR-200 family were found to be up-regulated in both primary and metastatic SOC. Interestingly, only two miRNAs (hsa-miR-1 and hsa-miR-3182) were down-regulated in both primary and metastatic SOC.

Comparing miRNA expression profiles in primary SOC (both chemo-sensitive and resistant/refractory) and omental metastases, 68 miRNAs were found to be up-regulated in both primary and metastatic SOC (Figure 5). Sixteen of these miRNAs were reported to be up-regulated in other studies (Table 4) [9, 13, 24, 25, 27], including all members of the miR-200 family. Interestingly, only two miRNAs (hsa-miR-1 and hsa-miR-3182) were down-regulated in both primary and metastatic SOC (Figure 5). Hsa-miR-1 was found to be significantly down-regulated in ovarian cancer compared to endometriosis by Wu *et al.* [10] whereas deregulation of hsa-miR-3182 in ovarian cancer has not been reported before. In addition, 87 miRNAs were up-regulated, and 75 miRNAs were down-regulated only in metastatic SOC (Figure 5 and Supplementary Table 7), which might play a crucial role in the metastasis of SOC.

**Table 4:** Aberrantly expressed core miRNAs in serous ovarian carcinoma

Up-regulated							
miRNA	Primary Resistant/Refractory		Primary Chemosensitive		Omental Metastases		Reference
	Fold Change^*^	*P*-value	Fold Change^*^	*P*-value	Fold Change&	*P*-value	
hsa-miR-200a-3p	16.32	4.35E-09	15.65	9.33E-09	12.38	1.66E-05	9, 13
hsa-miR-200b-3p	4.10	3.54E-03	2.73	3.77E-02	74.15	2.71E-12	13
hsa-miR-200c-3p	26.95	6.51E-10	24.13	2.63E-09	95.20	1.43E-09	13
hsa-miR-141-3p	18.57	1.68E-09	17.67	3.97E-09	6.10	9.39E-03	13
hsa-miR-429	84.48	8.15E-15	78.99	2.60E-14	31.00	1.75E-08	25
hsa-miR-203	331.38	1.10E-17	257.97	1.61E-16	28.07	1.18E-07	9
hsa-miR-205-5p	159.53	2.70E-08	181.37	1.90E-08	71.49	2.52E-05	9
hsa-miR-92a-3p	27.70	7.54E-10	33.82	1.71E-10	7.85	2.73E-10	9, 13
hsa-miR-214-3p	26.70	3.08E-06	16.36	6.45E-05	3.86	7.60E-04	24
hsa-miR-708-5p	15.09	7.52E-05	8.16	2.03E-03	2.14	7.08E-03	24
hsa-miR-23b-3p	14.69	1.95E-06	12.70	7.35E-06	2.23	1.09E-02	9, 13
hsa-miR-96-5p	13.43	1.25E-05	10.21	9.32E-05	4.23	4.85E-03	27
hsa-miR-93-5p	12.47	1.27E-12	10.52	2.53E-11	2.64	3.05E-03	9, 13
hsa-miR-20a-5p	12.27	8.07E-08	8.92	2.30E-06	3.96	6.69E-05	9, 13
hsa-miR-182-5p	9.97	2.18E-04	6.93	1.84E-03	6.35	2.85E-04	9, 13
hsa-miR-449a	4.71	2.00E-02	4.56	2.43E-02	166.60	5.17E-15	13
hsa-miR-425-5p	92.35	3.75E-16	86.45	1.26E-15	4.71	1.12E-06	
hsa-miR-500a-5p	85.26	2.61E-11	66.11	2.82E-10	3.66	1.62E-05	
hsa-miR-181c-5p	58.45	9.32E-09	24.74	3.42E-06	2.67	1.06E-02	
hsa-miR-423-5p	54.41	2.14E-12	34.86	2.07E-10	2.06	1.49E-02	
hsa-miR-454-3p	42.56	2.36E-07	20.01	2.66E-05	5.90	7.59E-07	
hsa-miR-345-5p	42.17	3.89E-10	35.29	2.51E-09	2.33	2.35E-03	
hsa-miR-30d-5p	32.53	5.37E-09	27.80	2.66E-08	3.25	2.77E-04	
hsa-miR-221-3p	30.49	8.65E-10	27.64	2.98E-09	2.76	9.18E-04	
hsa-miR-1301	29.67	1.91E-06	20.57	2.05E-05	7.06	1.55E-09	
hsa-miR-183-5p	26.35	4.41E-09	19.09	9.74E-08	7.80	1.98E-04	
hsa-miR-362-5p	22.71	2.86E-07	18.59	1.60E-06	3.10	5.11E-04	
hsa-miR-421	22.28	2.27E-06	12.59	9.60E-05	3.30	3.07E-05	
hsa-miR-130b-3p	22.26	2.07E-05	13.25	3.65E-04	5.08	7.93E-06	
hsa-miR-187-3p	21.07	1.53E-06	15.71	1.36E-05	42.60	3.87E-10	
hsa-miR-664-3p	19.96	3.20E-07	13.51	7.52E-06	3.76	1.74E-03	
hsa-miR-194-5p	18.61	4.58E-06	15.30	2.00E-05	2.48	6.55E-03	
hsa-miR-324-5p	17.09	2.57E-08	13.64	2.73E-07	2.14	1.08E-02	
hsa-miR-514a-3p	16.63	4.99E-03	33.81	5.98E-04	3.45	3.50E-02	
hsa-miR-155-5p	15.74	4.90E-03	7.00	4.69E-02	6.17	8.91E-07	
hsa-miR-26a-5p	14.93	5.94E-09	12.36	5.76E-08	2.27	2.61E-02	
hsa-miR-484	13.43	4.32E-08	10.59	5.79E-07	3.12	9.25E-05	
hsa-miR-941	13.12	1.11E-04	7.14	2.95E-03	5.40	1.74E-06	
hsa-miR-361-5p	12.67	1.51E-07	9.74	2.24E-06	3.14	1.21E-04	
hsa-miR-151a-5p	11.85	6.02E-06	9.75	3.18E-05	2.05	3.43E-02	
hsa-miR-342-3p	11.69	1.64E-08	9.55	1.99E-07	3.25	1.70E-04	
hsa-miR-92b-3p	11.43	4.98E-03	9.59	9.74E-03	18.81	8.49E-15	
hsa-miR-1275	11.29	2.94E-04	6.51	4.95E-03	2.85	2.00E-02	
hsa-miR-532-3p	11.18	7.47E-04	14.02	2.89E-04	3.68	1.55E-05	
hsa-miR-125a-3p	10.41	1.81E-07	8.63	1.54E-06	2.88	7.43E-04	
hsa-miR-1180	10.22	8.44E-05	7.95	4.62E-04	4.07	1.34E-06	
hsa-miR-18a-5p	10.02	2.02E-06	9.69	3.42E-06	2.71	3.66E-03	
hsa-miR-501-3p	9.11	3.07E-03	7.89	6.03E-03	2.37	2.86E-03	
hsa-miR-331-3p	8.62	6.53E-06	7.69	2.15E-05	3.79	2.61E-05	
hsa-miR-339-5p	8.05	9.04E-03	6.57	1.94E-02	2.84	1.89E-04	
hsa-miR-296-5p	8.01	3.28E-04	7.23	7.01E-04	17.64	2.53E-11	
hsa-miR-509-3-5p	7.18	2.68E-02	22.74	6.80E-04	9.48	1.37E-04	
hsa-miR-423-3p	7.06	2.84E-04	4.74	3.77E-03	2.37	1.82E-03	
hsa-miR-320b	6.44	1.64E-02	5.07	3.78E-02	2.89	1.16E-03	
hsa-miR-15b-5p	6.15	3.10E-06	6.28	3.13E-06	7.25	8.30E-10	
hsa-miR-1307-3p	6.05	1.56E-02	4.47	4.55E-02	5.34	3.62E-07	
hsa-miR-181b-5p	5.23	1.87E-05	4.64	7.54E-05	3.11	3.47E-05	
hsa-let-7c	4.78	1.72E-03	2.71	4.45E-02	2.53	3.95E-03	
hsa-miR-574-3p	4.63	1.30E-03	4.03	3.62E-03	2.08	8.88E-03	
hsa-miR-744-5p	4.59	4.09E-02	4.73	3.95E-02	4.59	2.14E-06	
hsa-miR-130a-3p	4.49	2.22E-04	3.62	1.55E-03	2.75	1.09E-03	
hsa-miR-107	4.14	2.12E-03	3.45	7.67E-03	4.28	7.57E-07	
hsa-miR-181a-5p	4.11	1.07E-03	2.61	2.54E-02	2.48	3.03E-03	
hsa-miR-210	3.80	1.14E-03	3.31	3.69E-03	4.08	1.42E-03	
hsa-miR-891a	3.60	1.49E-02	2.94	4.16E-02	46.68	3.15E-04	
hsa-miR-197-3p	3.36	1.67E-02	4.58	3.23E-03	2.21	5.81E-03	
hsa-miR-25-3p	2.79	6.06E-04	2.13	1.10E-02	2.12	8.46E-03	
hsa-miR-191-5p	2.59	6.59E-03	2.07	3.87E-02	4.61	7.59E-07	
**Down-regulated**							
hsa-miR-1	–4.09	2.89E-02	–7.19	2.84E-03	–18.76	8.17E-11	13
hsa-miR-3182	–9.71	9.77E-04	–10.80	6.68E-04	–3.27	4.41E-02	

^*^compared to normal fallopian tube; &compared to normal omentum.

### Validation of dysregulated core miRNAs

**Figure 6:**
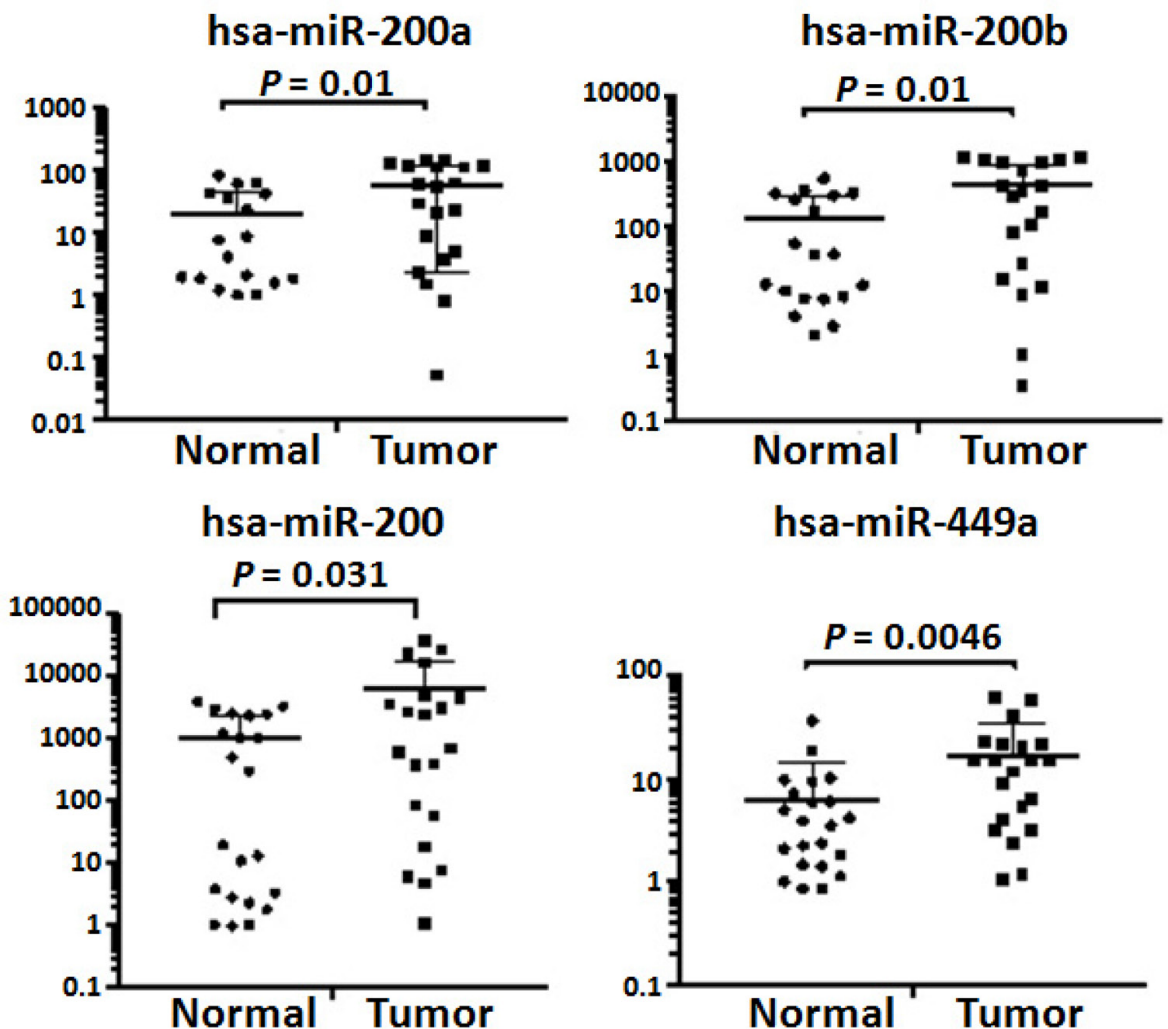
Validation of dysregulated miRNAs by quantitative RT-PCR Quantitative PCR was performed on 21 omental metastatic tissues and 22 normal omental tissues from 21 patients with SOC. Levels of microRNAs were normalized to miR-103-3p and expressed as fold change (2^–∆∆Ct^). Student’ *t*-test was used to compare means of two groups with a 95% confidence interval. There were five (4 normal and 1 tumor tissues) and four (3 normal and 1 tumor tissues) outliers for hsa-miR-200a and hsa-miR-200b, respectively. The expression levels of hsa-miR-200a and hsa-miR-200b in these outliers were either extremely low or extremely high, thus, were excluded from final calculations.

We used a relatively small cohort of samples for small RNA sequencing. To validate our findings, quantitative RT-PCR was performed on 22 normal omental tissues and 21 omenal metastatic tissues from 21 patients including 7 patients whose tissue samples were used for small RNA sequencing (Supplementary Table 1). All omental metastatic tissues were obtained from patients with high-grade SOC. We chose hsa-miR-200a, hsa-miR-200b, hsa-miR-200c and hsa-miR-449a since them are among miRNAs that were consistently shown to be up-regulated in this study (Table 4) and in several previously published studies [2, 13, 19, 25]. As expected, the quantitative RT-PCR results validated that hsa-miR-200a, hsa-miR-200b, hsa-miR-200c, and hsa-miR-449a were indeed up-regulated in omental metastases (Figure 6).

### Clinical relevant miRNAs in SOC

**Figure 7:**
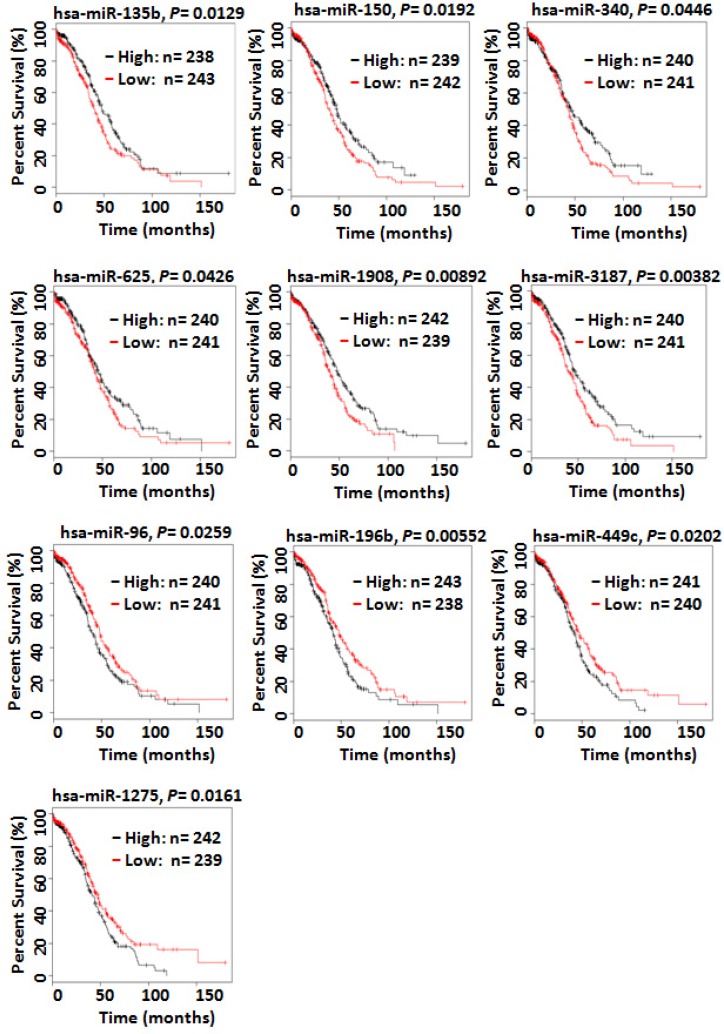
Kaplan–Meier overall survival curves for the ovarian cancer patient cohort in the TCGA data based on the expression of miRNAs Kaplan–Meier overall survival analysis indicated that high-level expression of hsa-miR-150 and hsa-miR-340 was associated with prolonged survival whereas high-level expression of hsa-miR-96, -196b, -449c, and -1275 was significantly correlated with a shorten overall survival. Hsa-miR-150 and hsa-miR-340 were down-regulated in omental metastases. Hsa-miR-96 and hsa-miR-1275 were up-regulated in both primary and metastatic SOC. Hsa-196b and hsa-miR-449c were only up-regulated in omental metastases.

To explore clinical associations of the miRNAs identified in this study to be dysregulated in either primary or metastatic SOC, we used the TCGA data that contains survival and miRNA expression data from 481 patients with high-grade SOC [28]. Kaplan–Meier overall survival analysis of all dysregulated miRNAs identified in this study indicated that high-level expression of hsa-miR-135, -150, -340, -652, -1908, and -miR-3187 was associated with prolonged survival whereas high-level expression of hsa-miR-96, -196b, -449c, and -1275 was significantly correlated with a shorten overall survival (Figure 7). Hsa-miR-150 and hsa-miR-340 were down-regulated in omental metastases. Hsa-652, -1908, and -3187 were down-regulated in both chemo-sensitive and chemo-resistant/refractory primary SOC whereas hsa-miR-135 was down-regulated only in chemo-sensitive primary SOC. Hsa-miR-96 and hsa-miR-1275 were up-regulated in both primary and metastatic SOC. Hsa-196b and hsa-miR-449c were up-regulated only in omental metastases.

## DISCUSSION

Using small RNA sequencing and annotation with the most recent version of miRbase (version 21, June 2017), we identified 251 miRNAs that were significantly differentially expressed in the omental metastasis of SOC compared with the normal omentum, a primary site of metastatic spread of ovarian carcinoma. These miRNAs may be useful in distinguishing malignant omentum from normal omentum. Furthermore, we determined the miRNA profiles in primary chemo-sensitive and chemo-resistant/refractory SOC using publicly available data. A total of 70 miRNAs were found to be aberrantly expressed (68 miRNAs up-regulated, 2 miRNAs down-regulated) in both primary (both chemo-sensitive and chemo-resistant/refractory) and metastatic SOC. Sixteen of these miRNAs were reported to be dysregulated in ovarian cancer in other studies (Table 4). This concordance strengthens our findings, supporting the relevance of these miRNAs to SOC.

The majority of patients with SOC die of metastatic disease, which remains poorly understood. Advanced metastatic disease often leads to recurrent and drug-resistant tumors. Vang *et al.* measured the expression of 377 miRNAs using qPCR arrays and identified 17 differentially expressed miRNAs (14 miRNAs up-regulated, 3 miRNAs down-regulated) in omental metastases compared to primary tumors [24]. We found a total of 251 miRNAs that were aberrantly expressed in omental metastases in comparison with the normal omentum using small RNA sequencing and annotation of 2588 mature human miRNAs. The overall miRNA expression clearly separated normal versus malignant omentum (Figure 1). Four miRNAs (hsa-miR-214, hsa-miR-708, hsa-let-7d, and hsa-miR-31) were up-regulated in both Vang *et al.* study and our study. Hsa-miR-214 and hsa-miR-708 were up-regulated in both primary and metastatic SOC, suggesting that these miRNAs may play an important role in tumorigenesis, growth, and metastasis. Indeed, hsa-miR-214 is deregulated in various types of tumors, acting as a key hub by coordinating several key signaling networks such as PTEN/AKT, β-catenin, and tyrosine kinase receptor pathways [29]. Specifically, hsa-miR-214 induces cell survival, cisplatin resistance and radioresistance in human ovarian cancer by targeting PTEN [20, 30]. Hsa-miR-708 acts as an oncogene in lung cancer by downregulating TMEM88 [31], but was reported to suppress ovarian cancer metastasis through targeting Rap1B [32]. Further studies are required to clarify the role of hsa-miR-708 in ovarian cancer. Recently, Samuel *et al.* reported that over-expression of hsa-miR-31 increased cisplatin resistance in ovarian cancer cells [33]. Hsa-let-7d is dysregulated in many types of cancer, acting as an either tumor suppressor or oncogene, its role in ovarian cancer has yet to be determined [34].

To complement our own small RNA sequencing data, we decided to analyze miRNA expression in primary SOC using publicly available data. We were able to identify the Patch2015 Nature study that included 7 normal fallopian samples and 80 primary tumor samples, of which 31 were chemo-sensitive tumors, 49 were resistant or refractory tumors [26]. The overall miRNA expression could clearly separate normal versus primary tumors except for one normal sample (Figure 3), however, could not distinguish chemo-sensitive tumors from resistant/refractory tumors. Further analysis indicated that 272 miRNAs were differentially expressed in both chemo-sensitive and resistant/refractory tumors (Figure 4). Seventy-four and 51 miRNAs were differentially expressed in only chemo-sensitive tumors and resistant/refractory tumors, respectively. Thus, although the overall miRNA expression patterns in chemo-sensitive and resistant/refractory tumors are alike, each tumor type has its own set of discrete miRNAs. Recently, Mihanfar *et al.* reviewed miRNAs involved in drug resistance in ovarian cancer and listed a total of 24 miRNAs that are involved in the development of multidrug resistance in ovarian cancer [35]. Fifteen of these 24 miRNAs were found to be up-regulated in primary resistant/refractory tumors in our study, validating our data reliability. Hsa-miR-188-5p and hsa-miR-631 were down-regulated only in primary resistant/refractory tumors. Hsa-miR-188-5p was shown to inhibit tumor cell proliferation and metastasis in hepatocellular carcinoma through targeting FGF5 [36], and in prostate cancer by repressing LAPTM4B expression [37]. Hsa-miR-631 inhibits the migration and invasion of prostate cancer cells by targeting ZAP70 [38] and resensitizes bortezomib-resistant multiple myeloma cells through the inhibition of UbcH10 [39]. The role of hsa-miR-188 and hsa-miR-631 in ovarian cancer remains undetermined.

We aimed to identify dysregulated core miRNAs in both primary and metastatic SOC, which might play a central role in tumorigenesis, growth, and metastasis. A total of 70 miRNAs were identified to be differentially expressed in both primary and metastatic SOC (Figure 5). The majority (68/70) of these miRNAs were up-regulated, only two miRNAs (hsa-miR-1 and hsa-miR-3182) were down-regulated. Sixteen of 68 up-regulated miRNAs were reported to be up-regulated in ovarian cancer in other studies (Table 4). Of note, all members of the miR-200 family were up-regulated. Most of the members of this family were found to be up-regulated in several previous studies [2, 19, 25]. Up-regulation of the miR-200 family might be caused by the amplification of the miRNA genes [40]. High levels of expression of miR-200 family in patients with SOC were shown to be correlated with a short overall survival [2]. Hsa-miR-1 is generally down-regulated and considered to be a tumor suppressor in many types of solid tumors. A recent study indicates that hsa-miR-1 inhibits the proliferation and migration of ovarian cancer cells by targeting the c-Met pathway, suggesting that hsa-miR-1 might act as a suppressor in ovarian cancer as well [41]. Dysregulation of hsa-miR-3182 in ovarian cancer has not been reported before. Its role in oncogenesis has yet to be elucidated.

Ten of the dysregulated miRNAs identified in this study were significantly associated with patient survival. In consistence with their expression patterns, high levels of expression of hsa-miR-135, -150, -340, -652, -1908, and -miR-3187 were correlated with prolonged survival whereas high levels of expression of hsa-miR-96, 196b, -449c, and -1275 decreased overall survival (Figure 7). Hsa-miR-150 has been reported to be down-regulated in human epithelial ovarian cancer and to inhibit cell invasion and metastasis through inhibiting transcriptional repressor ZEB1 [42]. Down-regulation of hsa-miR-150 is associated with aggressive clinicopathological features such as high clinical stage and pathological grade, short overall and progression-free survival. Although hsa-miR-340 is considered as a tumor suppressor in several solid tumors, its role in ovarian cancer is not well studied. A recent study, however, showed that hsa-miR-340 induced apoptosis and inhibited metastasis of ovarian cancer cells through inactivation of NF-κB1 [43]. Both hsa-miR-96 and hsa-miR196b were reported to be up-regulated in epithelial ovarian cancer while dysregulation of hsa-miR-449c and hsa-miR-1275 in ovarian cancer has not been reported before [27, 44]. Hsa-miR-196b promotes invasiveness of ovarian cancer cells through regulation of homeobox A9.

In summary, we performed a comprehensive miRNA expression analysis in the omental metastasis of SOC for the first time and identified 251 aberrantly expressed miRNAs that may be used to distinguish malignant omentum from normal omentum. Analysis of miRNA profiles in primary chemo-sensitive and chemo-resistant/refractory SOC indicates that while overall miRNA expression in chemo-sensitive and resistant/refractory tumors is quite similar, each tumor type has its own unique set of differentially expressed miRNAs, which may determine its sensitivity to chemotherapy. Comparing miRNA expression profiles in omental metastases and primary chemo-sensitive and chemo-resistant/refractory tumors, a set of 70 core miRNAs that were aberrantly expressed in both primary (both chemo-sensitive and chemo-resistant/refractory) and metastatic SOC has been identified for the first time. These aberrantly expressed core miRNAs may play crucial roles in tumorigenesis, growth, and metastasis. Therefore, they can serve as potential diagnostic biomarkers and as therapeutic targets for miRNA-mediated therapy. Kaplan–Meier overall survival analysis using TCGA data has demonstrated that ten miRNAs (hsa-miR-135, 150, -340, 625, 1908, 3187, -96, -196b, -449c, and -1275) are associated with survival of patients with SOC, which might serve as potential prognostic biomarkers.

## MATERIALS AND METHODS

### Human tissue samples

Normal and malignant omental tissues from patients with SOC were obtained in accordance with our protocol approved by the Institutional Review Board.

### RNA isolation

Human tissue samples were homogenized with a Bullet Blender 24 Gold (Next Advance, Inc., Troy, NY, USA). RNA was purified using miRNeasy Mini kit (Qiagen, Germantown, MD, USA) following the manufacturer’s instructions. The purity and concentration was determined on a NanoDrop 1000 spectrophotometer (Thermo Scientific, Wilmington, DE, USA).

### MiRNA library preparation and sequencing

500 ng of RNA was used for library preparation following the Illumina protocol with minor modifications. RNA library was made from each RNA sample by 3′ adapter ligation, 5′ RT primer annealing, 5′ adapter ligation, reverse transcription, and PCR amplification. Libraries were then pooled in batches of 12 samples in equal amounts and clustered with a concentration of 10.5 pmol in one lane each of a single read flowcell using the cBot (Illumina). Sequencing of 50 cycles was performed on a HiSeq 2500 (Illumina). Demultiplexing of the raw sequencing data and generation of the fastq files were done using CASAVA v.1.8.2.

### Sequencing data analysis

The sequencing data analysis was done using CLC Bio’s software, Biomedical Genomics Workbench v4.1.1 (Qiagen, Redwood City, CA, USA). Adapters were removed using cutadapt and aligned using a Smith-Waterman alignment and annotated using miRBase Release 21 and Homo_sapiens.GRCh38.ncrna (a list of *Homo sapiens* non-coding RNAs from ENSEMBL). The expression values were normalized using quantile normalization and transformed using log2. The expression values were then subject to differential gene expression analysis using “Exact Test” for two group comparison of normal vs tumor, etc. using edgeR package v 3.4.0 and the results were annotated using Ingenuity Pathway Analysis and enterprise TRANSFAC software.

### Public data analysis

We searched NCBI Gene Expression Omnibus for public available patient cohorts. Ovarian cancer studies without normal samples and with less than 50 tumor samples were filtered out to ensure statistical significance. We identified Patch2015 Nature study to include in our analysis [26]. MiRNA expression from GSE65819 were further log transformed in Partek^®^ software using the “MicroRNA Expression” workflow (Partek Inc., St. Louis, MO, USA.). Differentially expressed miRNAs were required to show a 2-fold difference in expression between normal and different response samples and an ANOVA contrast *p*-value meeting a threshold of *p*-value < 0.05. In total, 87 microarrays were analyzed using 2 ANOVA contrasts (refractory/resistant vs normal and sensitive vs normal). Venn diagrams were generated using Partek list manager to show overlap between different miRNA lists. To match different miRNAs alias in public data and our lab study, miRNA names are truncated to exclude the -1/-2 or -3p/-5p tails and followed by expert curation.

### Validation using quantitative RT-PCR

RNA from normal tissues and tumor samples was isolated as described above. Ten nanograms of RNA from each sample were used for cDNA synthesis using miRCURY LNA Universal microRNA PCR kit (Exiqon, Vedbaek, Denmark). The resulting cDNA from each sample was diluted 1:80 in RNase free water and 4 μL of the diluted cDNA was used for PCR amplification using miRCURY LNA Universal microRNA PCR kit following the manufacturer’s instructions. Levels of microRNAs of interest were normalized to miR-103-3p and expressed as fold change (2^–∆∆Ct^) as described by Livak and Schmittgen [45].

### Kaplan–Meier overall survival analysis

To determine the potential clinical significance of the miRNA signatures in SOC identified in this study, Kaplan–Meier overall survival analysis was performed on the cohort of 481 patients with high-grade SOC in the TCGA data (TCGA-OV, https://portal.gdc.cancer.gov/projects/TCGA-OV). The “high” and “low” groups were segregated based on median miRNA expression values. Kaplan–Meier survival analysis was used to determine the survival differences between “high” and “low” miRNA expression groups, which were visualized by Kaplan–Meier plots and compared using Cox regression analysis, with *P*-values calculated by log-rank test using the Survival package in R [46]. The survival differences were considered to be statistically significant when *P*-values were < 0.05.

## SUPPLEMENTARY MATERIALS TABLES












