# Supplementary material for: Identification of core aberrantly expressed microRNAs in serous ovarian carcinoma
Source: Oncotarget. 2018 Apr 17;9(29):20451–66. doi: 10.18632/oncotarget.24942 (PMC5945511; doi:10.18632/oncotarget.24942)
Supplement: Supplementary file 1 [file oncotarget-09-20451-s001.pdf]

Identification of core aberrantly expressed microRNAs in serous ovarian carcinoma

SUPPLEMENTARY MATERIALS

Supplementary Table 1: Demographic characteristics of the patients participating in the study

Characteristics	Small RNA Sequencing (7 patients)	Independent Validation (21 patients)
**Age, median (range)**	60 (42–71)	60 (41–85)
**Race (%)**		
Caucasian	7 (100)	21 (100)
**Histology (%)**		
Serous	6 (85.7)	20 (95.2)
Anaplastic carcinoma	1 (14.3)	1 (4.8)
**Grade (%)**		
3	7 (100)	19 (90.5)
4		1 (4.8)
unknown		1 (4.8)

Supplementary Table 2: Annotation statistics of small RNA sequencing data

Sample ID	Total # of Reads	Avg Length of Reads	Range of Lengths	Most Frequent Length of Read	Total # of Annotated Reads	Reads Annotated with miRBase (Homo Sapiens)	% of Reads Annotated with miRBase (Homo Sapiens)
T1	31,085,803	31.5	18–48	32	8,526,981	169,307	1.99
T2	30,718,755	30.3	16–48	31	8,670,252	1269471	14.64
T3	32,633,086	28.8	16–48	32	10,190,714	2864534	28.11
T4	30,238,102	27.6	16–48	32	9,905,439	3578207	36.12
T5	33,909,390	23.8	16–48	22	18,784,911	10,181,198	54.20
T6	27,599,431	29.1	16–48	22	13,526,110	6,205,753	45.88
T7	25,056,756	24.7	16–48	16	6,331,334	1,214,873	19.19
T8	28,887,709	22.9	16–48	22	13,089,777	7,343,132	56.10
T9	35,793,937	28.3	16–48	33	16,803,163	6,722,415	40.01
N1	20,872,784	24.0	16–48	16	6,848,487	1546817	22.59
N2	22,701,451	25.3	16–48	16	6,831,604	1,554,129	22.75
N3	29,536,471	25.7	16–48	22	11,758,055	5,000,646	42.53
N4	29,219,780	25.5	16–48	22	11,483,422	5,226,079	45.51
N5	23,207,707	26.3	16–48	22	8,024,114	2,058,017	25.65
N6	24,693,934	25.1	16–48	22	9,169,688	3,945,148	43.02
N7	24,077,085	26.9	16–48	22	8,646,708	3,405,788	39.39
N8	23,986,301	24.9	16–48	22	8,636,773	3,680,816	42.62
N9	21,885,189	24.2	16–48	16	6,592,928	1,873,628	28.42
All tumor samples	275,922,969	27.5	16–48	32	105,828,681	39,548,890	37.37
All normal samples	220,180,702	25	16–48	22	77,991,779	28,291,068	36.27
All samples	496,103,671	27	16–48	22	183,820,460	67,839,958	36.91

T1–T9 were omental metastatic tissue samples, N1–N9 were normal omental tissue samples.

Supplementary Table 3: Aberrantly expressed miRNAs in omental metastases. See Supplementary_Table_3

Supplementary Table 4: Aberrantly expressed miRNAs in primary chemo-sensitive tumors. See Supplementary_Table_4

Supplementary Table 5: Aberrantly expressed miRNAs in primary resistant/refractory tumors. See Supplementary_Table_5

Supplementary Table 6: Aberrantly expressed miRNAs only in primary resistant/refractory tumors, but not in primary chemo-sensitive tumors. See Supplementary_Table_6

Supplementary Table 7: Aberrantly expressed miRNAs only in omental metastases, but not in primary tumors. See Supplementary_Table_7
