# Supplementary material for: Identification of core aberrantly expressed microRNAs in serous ovarian carcinoma
Source: Oncotarget. 2018 Apr 17;9(29):20451–66. doi: 10.18632/oncotarget.24942 (PMC5945511; doi:10.18632/oncotarget.24942)
Supplement: Supplementary file 2 [file oncotarget-09-20451-s002.docx]

**Supplementary Table 3.** Aberrantly expressed miRNAs in omental metastases

| Up-regulated | | | Down-regulated | | |
| --- | --- | --- | --- | --- | --- |
| Name | Fold Change$ | *P*-value | Name | Fold Change$ | *P*-value |
| hsa-miR-449c-5p | 1914.70 | 2.40E-17 | hsa-miR-5683-5p | -272.43 | 6.93E-24 |
| hsa-miR-885-3p | 268.22 | 4.77E-13 | hsa-miR-216a-5p | -30.21 | 2.34E-09 |
| hsa-miR-1269a-3p | 170.82 | 4.66E-09 | hsa-miR-144-3p | -23.95 | 1.16E-05 |
| hsa-miR-449a-5p# | 166.60 | 5.17E-15 | hsa-miR-1-2-3p# | -18.76 | 8.17E-11 |
| hsa-miR-885-5p | 151.76 | 3.04E-19 | hsa-miR-143-3p | -16.77 | 3.38E-13 |
| hsa-miR-6510-3p | 151.48 | 5.84E-06 | hsa-miR-215-5p | -11.81 | 1.09E-07 |
| hsa-miR-200c-3p# | 95.20 | 1.43E-09 | hsa-miR-217-5p | -11.63 | 2.80E-07 |
| hsa-miR-200b-3p# | 74.15 | 2.71E-12 | hsa-miR-126-5p | -8.83 | 3.17E-07 |
| hsa-miR-205-5p* | 71.49 | 2.52E-05 | hsa-miR-451a-5p# | -8.53 | 9.45E-06 |
| hsa-miR-200b-5p | 65.77 | 5.76E-11 | hsa-miR-122-5p | -8.45 | 1.49E-02 |
| hsa-miR-375-3p# | 50.38 | 4.45E-09 | hsa-miR-511-3p | -8.21 | 8.89E-08 |
| hsa-miR-891a-5p | 46.68 | 3.15E-04 | hsa-miR-144-5p | -7.95 | 1.41E-04 |
| hsa-miR-187-3p | 42.60 | 3.87E-10 | hsa-miR-376a-1-5p | -7.70 | 2.55E-07 |
| hsa-miR-429-3p | 31.00 | 1.75E-08 | hsa-miR-374a-3p | -7.46 | 5.68E-05 |
| hsa-miR-203b-3p | 28.63 | 3.07E-09 | hsa-miR-542-3p | -7.21 | 1.25E-06 |
| hsa-miR-4697-3p | 28.08 | 2.06E-06 | hsa-miR-143-5p | -6.97 | 9.76E-09 |
| hsa-miR-203a-3p* | 28.07 | 1.18E-07 | hsa-miR-142-3p | -6.85 | 2.10E-04 |
| hsa-miR-449b-5p | 25.81 | 5.58E-07 | hsa-miR-5701-1-5p | -6.82 | 1.43E-03 |
| hsa-miR-135b-3p | 25.58 | 1.11E-11 | hsa-miR-424-5p | -6.77 | 6.80E-08 |
| hsa-miR-183-3p | 20.71 | 7.94E-07 | hsa-miR-126-3p | -6.65 | 5.93E-08 |
| hsa-miR-135b-5p | 19.40 | 2.86E-09 | hsa-miR-3611-3p | -6.60 | 2.28E-04 |
| hsa-miR-92b-3p | 18.81 | 8.49E-15 | hsa-miR-139-3p | -6.47 | 6.67E-06 |
| hsa-miR-296-5p | 17.64 | 2.53E-11 | hsa-miR-136-3p | -6.27 | 1.40E-04 |
| hsa-miR-200a-5p | 16.58 | 1.33E-05 | hsa-miR-497-5p | -6.13 | 9.74E-07 |
| hsa-miR-200c-5p | 16.19 | 1.38E-05 | hsa-miR-4454-5p | -6.12 | 1.13E-05 |
| hsa-miR-1292-5p | 14.32 | 2.17E-07 | hsa-miR-145-3p | -5.87 | 2.76E-07 |
| hsa-miR-135a-1-5p | 13.14 | 9.55E-04 | hsa-miR-29c-3p | -5.68 | 3.90E-06 |
| hsa-miR-1226-3p | 12.70 | 2.21E-11 | hsa-miR-618-5p | -5.62 | 1.65E-07 |
| hsa-miR-200a-3p*# | 12.38 | 1.66E-05 | hsa-miR-133a-1-3p# | -5.54 | 4.52E-06 |
| hsa-miR-577-5p | 12.19 | 2.80E-05 | hsa-miR-138-2-5p | -5.49 | 3.72E-04 |
| hsa-miR-31-5p | 12.02 | 1.77E-10 | hsa-miR-140-5p | -5.46 | 8.24E-08 |
| hsa-miR-625-3p | 10.10 | 1.34E-10 | hsa-miR-1973-3p | -5.43 | 3.34E-04 |
| hsa-miR-181d-5p | 9.74 | 1.21E-08 | hsa-miR-190a-5p | -5.15 | 2.21E-05 |
| hsa-miR-3200-3p | 9.61 | 3.59E-09 | hsa-miR-7641-1-5p | -5.12 | 8.53E-04 |
| hsa-miR-509-3-5p | 9.48 | 1.37E-04 | hsa-miR-139-5p | -4.99 | 9.43E-07 |
| hsa-miR-509-1-3p | 9.36 | 3.01E-06 | hsa-miR-374a-5p | -4.99 | 4.83E-04 |
| hsa-miR-1270-5p | 9.13 | 3.48E-05 | hsa-miR-4792-5p | -4.84 | 2.96E-03 |
| hsa-miR-548az-5p | 9.01 | 9.03E-04 | hsa-miR-193a-3p | -4.79 | 2.02E-04 |
| hsa-miR-92a-1-3p*# | 7.85 | 2.73E-10 | hsa-miR-3653-3p | -4.76 | 1.86E-03 |
| hsa-miR-522-3p | 7.83 | 3.22E-04 | hsa-miR-582-3p | -4.72 | 2.59E-04 |
| hsa-miR-183-5p | 7.80 | 1.98E-04 | hsa-miR-142-5p | -4.65 | 1.34E-04 |
| hsa-mirR7706-3p | 7.79 | 2.21E-07 | hsa-miR-101-1-3p | -4.59 | 5.81E-04 |
| hsa-miR-877-3p | 7.40 | 1.20E-06 | hsa-miR-455-5p | -4.32 | 3.73E-05 |
| hsa-miR-92b-5p | 7.37 | 4.29E-06 | hsa-miR-887-3p | -4.28 | 4.95E-04 |
| hsa-miR-15b-5p | 7.25 | 8.30E-10 | hsa-miR-335-5p | -4.23 | 9.15E-06 |
| hsa-miR-508-3p | 7.07 | 1.66E-04 | hsa-miR-377-3p | -4.12 | 1.06E-04 |
| hsa-miR-1301-3p | 7.06 | 1.55E-09 | hsa-miR-29c-5p | -3.93 | 1.48E-05 |
| hsa-miR-18a-3p | 6.87 | 6.51E-08 | hsa-miR-450b-5p | -3.63 | 1.54E-03 |
| hsa-let-7d-3p | 6.81 | 7.91E-10 | hsa-miR-337-5p | -3.62 | 4.46E-04 |
| hsa-miR-296-3p | 6.78 | 5.04E-07 | hsa-miR-195a-5p | -3.58 | 5.30E-04 |
| hsa-miR-92a-1-5p | 6.75 | 8.15E-05 | hsa-miR-199b-5p | -3.55 | 8.68E-04 |
| hsa-miR-1303-3p | 6.54 | 4.53E-08 | hsa-miR-675-3p | -3.54 | 1.22E-04 |
| hsa-miR-3687-1-3p | 6.53 | 3.57E-06 | hsa-miR-6087-5p | -3.50 | 2.03E-03 |
| hsa-miR-1306-5p | 6.48 | 4.51E-07 | hsa-miR-3196-5p | -3.49 | 6.85E-03 |
| hsa-miR-182-5p*# | 6.35 | 2.85E-04 | hsa-miR-7704-5p | -3.40 | 6.92E-03 |
| hsa-miR-25-5p | 6.34 | 1.39E-04 | hsa-miR-140-3p | -3.33 | 1.55E-05 |
| hsa-miR-155-5p | 6.17 | 8.91E-07 | hsa-miR-3182-5p | -3.27 | 4.41E-02 |
| hsa-miR-141-3p# | 6.10 | 9.39E-03 | hsa-miR-376c-3p | -3.23 | 1.69E-03 |
| hsa-miR-454-3p | 5.90 | 7.59E-07 | hsa-miR-452-5p | -3.22 | 1.40E-03 |
| hsa-miR-3065-5p | 5.42 | 1.20E-06 | hsa-miR-338-3p | -3.18 | 1.87E-02 |
| hsa-miR-941-1-3p | 5.40 | 1.74E-06 | hsa-miR-450a-1-5p | -3.14 | 1.30E-03 |
| hsa-miR-1307-3p | 5.34 | 3.62E-07 | hsa-miR-4492-3p | -3.00 | 2.14E-02 |
| hsa-miR-760-3p | 5.20 | 3.64E-04 | hsa-miR-1246-5p | -2.96 | 7.70E-03 |
| hsa-miR-1291-5p | 5.20 | 3.35E-06 | hsa-miR-4516-5p | -2.93 | 9.99E-03 |
| hsa-miR-3065-3p | 5.12 | 9.24E-06 | hsa-miR-495-3p | -2.93 | 2.52E-03 |
| hsa-miR-130b-3p | 5.08 | 7.93E-06 | hsa-miR-199a-1-3p | -2.83 | 3.06E-03 |
| hsa-miR-877-5p | 5.03 | 1.45E-05 | hsa-miR-152-3p* | -2.82 | 9.20E-04 |
| hsa-miR-15b-3p | 4.94 | 2.11E-04 | hsa-miR-376a-1-3p | -2.80 | 4.34E-03 |
| hsa-miR-103a-2-3p | 4.92 | 6.55E-08 | hsa-miR-7977-5p | -2.74 | 1.78E-02 |
| hsa-miR-149-5p | 4.82 | 2.36E-06 | hsa-miR-3195-5p | -2.65 | 3.56E-02 |
| hsa-miR-141-5p | 4.81 | 4.06E-02 | hsa-miR-381-3p | -2.61 | 8.64E-03 |
| hsa-miR-3620-3p | 4.72 | 2.78E-06 | hsa-miR-379-5p | -2.60 | 3.99E-03 |
| hsa-miR-425-5p | 4.71 | 1.12E-06 | hsa-miR-127-5p | -2.58 | 2.04E-03 |
| hsa-miR-191-5p | 4.61 | 7.59E-07 | hsa-miR-337-3p | -2.53 | 1.76E-02 |
| hsa-miR-34c-3p | 4.61 | 2.00E-03 | hsa-miR-2355-3p | -2.53 | 7.10E-04 |
| hsa-miR-744-5p | 4.59 | 2.14E-06 | hsa-miR-486-1-5p | -2.52 | 2.08E-02 |
| hsa-miR-125b-1-3p | 4.54 | 5.40E-04 | hsa-miR-150-5p | -2.44 | 1.32E-02 |
| hsa-miR-125a-5p | 4.48 | 2.05E-05 | hsa-miR-22-3p* | -2.37 | 5.88E-03 |
| hsa-miR-99b-5p | 4.47 | 2.20E-06 | hsa-miR-340-5p | -2.35 | 3.12E-02 |
| hsa-miR-625-5p | 4.42 | 9.06E-06 | hsa-miR-29a-5p | -2.34 | 1.54E-02 |
| hsa-miR-17-5p# | 4.38 | 4.25E-06 | hsa-miR-145a-5p | -2.18 | 2.11E-02 |
| hsa-miR-107-3p | 4.28 | 7.57E-07 | hsa-miR-584-5p | -2.17 | 4.75E-03 |
| hsa-miR-125b-1-5p | 4.26 | 7.74E-04 | hsa-let-7i-3p | -2.14 | 2.18E-02 |
| hsa-miR-96-5p | 4.23 | 4.85E-03 | hsa-miR-9-1-5p | -2.09 | 2.97E-02 |
| hsa-miR-501-5p | 4.22 | 1.68E-06 |  |  |  |
| hsa-miR-210-3p | 4.08 | 1.42E-03 |  |  |  |
| hsa-miR-1180-3p | 4.07 | 1.34E-06 |  |  |  |
| hsa-miR-6501-5p | 4.04 | 1.36E-03 |  |  |  |
| hsa-miR-2110-5p | 3.98 | 1.93E-05 |  |  |  |
| hsa-miR-20a-5p*# | 3.96 | 6.69E-05 |  |  |  |
| hsa-miR-16-2-3p | 3.88 | 4.11E-04 |  |  |  |
| hsa-miR-214-3p | 3.86 | 7.60E-04 |  |  |  |
| hsa-miR-361-3p | 3.82 | 4.52E-05 |  |  |  |
| hsa-miR-331-3p | 3.79 | 2.61E-05 |  |  |  |
| hsa-miR-664a-3p | 3.76 | 1.74E-03 |  |  |  |
| hsa-let-7e-5p | 3.70 | 4.64E-06 |  |  |  |
| hsa-miR-532-3p | 3.68 | 1.55E-05 |  |  |  |
| hsa-miR-500a-5p | 3.66 | 1.62E-05 |  |  |  |
| hsa-miR-671-3p | 3.55 | 2.04E-04 |  |  |  |
| hsa-miR-129-1-5p | 3.45 | 7.54E-03 |  |  |  |
| hsa-miR-514a-1-3p | 3.45 | 3.50E-02 |  |  |  |
| hsa-miR-331-5p | 3.42 | 7.87E-05 |  |  |  |
| hsa-miR-421-3p | 3.30 | 3.07E-05 |  |  |  |
| hsa-miR-196b-5p# | 3.25 | 7.23E-03 |  |  |  |
| hsa-miR-30d-5p | 3.25 | 2.77E-04 |  |  |  |
| hsa-miR-342-3p | 3.25 | 1.70E-04 |  |  |  |
| hsa-miR-23b-5p | 3.23 | 1.55E-04 |  |  |  |
| hsa-miR-361-5p | 3.14 | 1.21E-04 |  |  |  |
| hsa-miR-484-5p | 3.12 | 9.25E-05 |  |  |  |
| hsa-miR-181b-1-5p | 3.11 | 3.47E-05 |  |  |  |
| hsa-miR-362-5p | 3.10 | 5.11E-04 |  |  |  |
| hsa-miR-93-3p | 3.07 | 6.51E-04 |  |  |  |
| hsa-miR-320b-1-3p | 2.89 | 1.16E-03 |  |  |  |
| hsa-miR-125a-3p | 2.88 | 7.43E-04 |  |  |  |
| hsa-miR-485-3p | 2.86 | 5.65E-03 |  |  |  |
| hsa-miR-769-3p | 2.86 | 1.34E-03 |  |  |  |
| hsa-miR-1275-5p | 2.85 | 2.00E-02 |  |  |  |
| hsa-miR-339-5p | 2.84 | 1.89E-04 |  |  |  |
| hsa-miR-1249-3p | 2.83 | 3.63E-03 |  |  |  |
| hsa-miR-222-3p | 2.82 | 2.73E-03 |  |  |  |
| hsa-miR-221-3p | 2.76 | 9.18E-04 |  |  |  |
| hsa-miR-3615-3p | 2.75 | 6.25E-03 |  |  |  |
| hsa-miR-130a-3p | 2.75 | 1.09E-03 |  |  |  |
| hsa-miR-4443-5p | 2.74 | 2.02E-02 |  |  |  |
| hsa-let-7d-5p | 2.73 | 4.42E-04 |  |  |  |
| hsa-miR-330-3p | 2.73 | 1.04E-03 |  |  |  |
| hsa-miR-18a-5p | 2.71 | 3.66E-03 |  |  |  |
| hsa-miR-433-3p | 2.67 | 2.84E-02 |  |  |  |
| hsa-miR-181c-5p | 2.67 | 1.06E-02 |  |  |  |
| hsa-miR-106b-3p | 2.67 | 4.93E-03 |  |  |  |
| hsa-miR-93-5p*# | 2.64 | 3.05E-03 |  |  |  |
| hsa-miR-425-3p | 2.63 | 8.25E-04 |  |  |  |
| hsa-miR-664b-3p | 2.62 | 2.61E-02 |  |  |  |
| hsa-miR-99b-3p | 2.58 | 5.31E-04 |  |  |  |
| hsa-miR-532-5p | 2.56 | 3.19E-03 |  |  |  |
| hsa-miR-6511a-1-3p | 2.55 | 1.01E-02 |  |  |  |
| hsa-miR-342-5p | 2.54 | 2.42E-03 |  |  |  |
| hsa-let-7c-5p | 2.53 | 3.95E-03 |  |  |  |
| hsa-miR-181a-2-5p | 2.48 | 3.03E-03 |  |  |  |
| hsa-miR-194-1-5p | 2.48 | 6.55E-03 |  |  |  |
| hsa-miR-766-3p | 2.47 | 4.03E-03 |  |  |  |
| hsa-miR-423-3p | 2.37 | 1.82E-03 |  |  |  |
| hsa-miR-550a-1-3p | 2.37 | 1.76E-02 |  |  |  |
| hsa-miR-320c-1-3p | 2.37 | 1.43E-02 |  |  |  |
| hsa-miR-501-3p | 2.37 | 2.86E-03 |  |  |  |
| hsa-miR-345-5p | 2.33 | 2.35E-03 |  |  |  |
| hsa-miR-26a-1-5p | 2.27 | 2.61E-02 |  |  |  |
| hsa-miR-132-3p | 2.24 | 6.81E-03 |  |  |  |
| hsa-miR-23b-3p*# | 2.23 | 1.09E-02 |  |  |  |
| hsa-miR-128-1-3p | 2.22 | 2.72E-03 |  |  |  |
| hsa-miR-197-3p | 2.21 | 5.81E-03 |  |  |  |
| hsa-miR-224-5p | 2.18 | 4.47E-02 |  |  |  |
| hsa-miR-192-5p | 2.17 | 2.90E-02 |  |  |  |
| hsa-miR-3928-3p | 2.15 | 1.14E-02 |  |  |  |
| hsa-miR-708-5p | 2.14 | 7.08E-03 |  |  |  |
| hsa-miR-324-5p | 2.14 | 1.08E-02 |  |  |  |
| hsa-miR-30b-3p | 2.12 | 1.54E-02 |  |  |  |
| hsa-miR-25-3p | 2.12 | 8.46E-03 |  |  |  |
| hsa-miR-320a-3p | 2.11 | 9.00E-03 |  |  |  |
| hsa-miR-125b-2-3p | 2.09 | 2.83E-02 |  |  |  |
| hsa-miR-574-3p | 2.08 | 8.88E-03 |  |  |  |
| hsa-let-7e-3p | 2.06 | 1.24E-02 |  |  |  |
| hsa-miR-423-5p | 2.06 | 1.49E-02 |  |  |  |
| hsa-miR-1296-5p | 2.05 | 1.12E-02 |  |  |  |
| hsa-miR-151a-5p | 2.05 | 3.43E-02 |  |  |  |
| hsa-miR-103a-2-5p | 2.04 | 2.52E-02 |  |  |  |
| hsa-miR-500a-3p | 2.03 | 1.78E-02 |  |  |  |

* concordant with reference 9; # concordant with reference 13; $ compared to normal omentum.
