# Supplementary material for: Identification of core aberrantly expressed microRNAs in serous ovarian carcinoma
Source: Oncotarget. 2018 Apr 17;9(29):20451–66. doi: 10.18632/oncotarget.24942 (PMC5945511; doi:10.18632/oncotarget.24942)
Supplement: Supplementary file 3 [file oncotarget-09-20451-s003.docx]

**Supplementary Table 4.** Aberrantly expressed miRNAs in primary chemo-sensitive tumors

| Up-regulated | | | Down-regulated | | |
| --- | --- | --- | --- | --- | --- |
| Name | Fold Change* | *P*-value | Name | Fold Change* | *P*-value |
| hsa-miR-203 | 257.97 | 1.61E-16 | hsa-miR-767-5p | -19.26 | 1.58E-03 |
| hsa-miR-143-3p | 195.06 | 1.12E-07 | hsa-miR-486-3p | -14.13 | 9.03E-04 |
| hsa-miR-205-5p | 181.37 | 1.90E-08 | hsa-miR-646 | -13.95 | 1.15E-03 |
| hsa-miR-150-5p | 152.45 | 2.66E-08 | hsa-miR-938 | -13.33 | 1.70E-03 |
| hsa-miR-451a | 138.28 | 1.07E-08 | hsa-miR-562 | -10.88 | 2.80E-03 |
| hsa-miR-199a-3p | 117.62 | 1.63E-09 | hsa-miR-3182 | -10.80 | 6.68E-04 |
| hsa-miR-199b-5p | 106.57 | 4.64E-09 | hsa-miR-1248 | -10.59 | 1.31E-03 |
| hsa-miR-223-3p | 92.67 | 6.86E-11 | hsa-miR-302c-3p | -10.46 | 2.48E-03 |
| hsa-miR-4286 | 91.23 | 4.85E-09 | hsa-miR-515-5p | -10.39 | 2.01E-03 |
| hsa-miR-425-5p | 86.45 | 1.26E-15 | hsa-miR-339-3p | -10.24 | 1.03E-03 |
| hsa-miR-429 | 78.99 | 2.60E-14 | hsa-miR-586 | -10.20 | 2.84E-03 |
| hsa-miR-513b | 67.71 | 4.36E-04 | hsa-miR-548ak | -9.91 | 2.59E-03 |
| hsa-miR-500a-5p | 66.11 | 2.82E-10 | hsa-miR-1288 | -9.90 | 4.27E-03 |
| hsa-miR-1260b | 56.98 | 1.62E-10 | hsa-miR-625-5p | -9.73 | 6.87E-04 |
| hsa-miR-30c-5p | 56.52 | 2.62E-10 | hsa-miR-550a-5p | -9.23 | 5.59E-03 |
| hsa-miR-27a-3p | 47.00 | 5.53E-10 | hsa-miR-580 | -9.11 | 2.89E-03 |
| hsa-miR-1915-3p | 38.35 | 6.80E-07 | hsa-miR-518a-3p | -9.11 | 2.27E-03 |
| hsa-miR-575 | 36.05 | 1.82E-07 | hsa-miR-3136-5p | -9.10 | 2.65E-03 |
| hsa-miR-345-5p | 35.29 | 2.51E-09 | hsa-miR-127-5p | -8.89 | 6.33E-03 |
| hsa-miR-1246 | 35.14 | 1.40E-02 | hsa-miR-450b-3p | -8.43 | 1.42E-03 |
| hsa-miR-106a-5p | 35.07 | 1.27E-16 | hsa-miR-576-5p | -8.37 | 1.16E-02 |
| hsa-miR-423-5p | 34.86 | 2.07E-10 | hsa-miR-1257 | -8.21 | 1.47E-03 |
| hsa-miR-513c-5p | 34.82 | 3.55E-04 | hsa-miR-520c-3p | -8.07 | 5.74E-03 |
| hsa-miR-92a-3p | 33.82 | 1.71E-10 | hsa-miR-1258 | -7.93 | 7.05E-03 |
| hsa-miR-514a-3p | 33.81 | 5.98E-04 | hsa-miR-568 | -7.86 | 1.27E-02 |
| hsa-miR-10b-5p | 33.62 | 4.98E-10 | hsa-miR-1205 | -7.84 | 7.82E-03 |
| hsa-miR-340-5p | 30.43 | 7.53E-11 | hsa-miR-802 | -7.58 | 1.48E-02 |
| hsa-miR-30d-5p | 27.80 | 2.66E-08 | hsa-miR-576-3p | -7.55 | 1.05E-02 |
| hsa-miR-221-3p | 27.64 | 2.98E-09 | hsa-miR-2277-3p | -7.45 | 4.90E-03 |
| hsa-miR-660-5p | 27.05 | 3.96E-07 | hsa-miR-515-3p | -7.29 | 9.47E-03 |
| hsa-miR-720 | 26.58 | 1.60E-07 | hsa-miR-1 | -7.19 | 2.84E-03 |
| hsa-miR-142-3p | 26.44 | 2.58E-07 | hsa-miR-548d-5p | -7.16 | 6.84E-03 |
| hsa-miR-1260a | 26.34 | 1.91E-10 | hsa-miR-3187-3p | -7.11 | 1.95E-03 |
| hsa-miR-509-3p | 25.35 | 6.58E-04 | hsa-miR-762 | -7.04 | 4.93E-03 |
| hsa-miR-181c-5p | 24.74 | 3.42E-06 | hsa-miR-367-3p | -7.01 | 2.24E-03 |
| hsa-miR-99a-5p | 24.26 | 2.77E-07 | hsa-miR-3175 | -6.91 | 9.87E-04 |
| hsa-miR-200c-3p | 24.13 | 2.63E-09 | hsa-miR-1245b-5p | -6.86 | 3.98E-03 |
| hsa-miR-30b-5p | 23.34 | 3.73E-08 | hsa-miR-3605-5p | -6.83 | 4.57E-03 |
| hsa-miR-509-3-5p | 22.74 | 6.80E-04 | hsa-miR-890 | -6.70 | 6.62E-03 |
| hsa-miR-1301 | 20.57 | 2.05E-05 | hsa-miR-3141 | -6.62 | 1.27E-02 |
| hsa-miR-320e | 20.44 | 5.65E-07 | hsa-miR-217 | -6.39 | 1.91E-02 |
| hsa-miR-617 | 20.40 | 1.53E-04 | hsa-miR-1324 | -6.35 | 1.06E-03 |
| hsa-miR-454-3p | 20.01 | 2.66E-05 | hsa-miR-370 | -6.30 | 7.03E-03 |
| hsa-miR-183-5p | 19.09 | 9.74E-08 | hsa-miR-302e | -6.30 | 8.85E-03 |
| hsa-miR-362-5p | 18.59 | 1.60E-06 | hsa-miR-4431 | -6.28 | 2.52E-03 |
| hsa-miR-126-3p | 18.19 | 1.67E-10 | hsa-miR-369-3p | -6.24 | 2.91E-02 |
| hsa-miR-141-3p | 17.67 | 3.97E-09 | hsa-miR-519c-3p | -6.15 | 2.39E-02 |
| hsa-miR-199a-5p | 17.46 | 1.26E-05 | hsa-miR-563 | -6.14 | 1.53E-02 |
| hsa-miR-24-3p | 17.25 | 1.96E-08 | hsa-miR-548an | -6.12 | 4.04E-03 |
| hsa-miR-146b-5p | 17.17 | 2.08E-05 | hsa-miR-23c | -6.09 | 1.75E-02 |
| hsa-miR-4516 | 16.89 | 6.60E-07 | hsa-miR-1183 | -6.01 | 1.69E-02 |
| hsa-miR-378d | 16.37 | 9.25E-04 | hsa-miR-544a | -6.00 | 1.62E-03 |
| hsa-miR-214-3p | 16.36 | 6.45E-05 | hsa-miR-499a-3p | -5.90 | 1.12E-02 |
| hsa-miR-596 | 16.09 | 3.44E-05 | hsa-miR-4458 | -5.89 | 8.17E-04 |
| hsa-miR-187-3p | 15.71 | 1.36E-05 | hsa-miR-1908 | -5.87 | 1.51E-03 |
| hsa-miR-200a-3p | 15.65 | 9.33E-09 | hsa-miR-606 | -5.86 | 2.48E-02 |
| hsa-miR-455-3p | 15.35 | 5.26E-04 | hsa-miR-485-5p | -5.85 | 1.35E-02 |
| hsa-miR-194-5p | 15.30 | 2.00E-05 | hsa-miR-761 | -5.79 | 2.81E-03 |
| hsa-miR-532-3p | 14.02 | 2.89E-04 | hsa-miR-1825 | -5.77 | 1.48E-04 |
| hsa-miR-520h | 13.98 | 9.26E-05 | hsa-miR-3168 | -5.74 | 1.84E-03 |
| hsa-miR-362-3p | 13.70 | 8.43E-05 | hsa-miR-1284 | -5.71 | 7.03E-03 |
| hsa-miR-324-5p | 13.64 | 2.73E-07 | hsa-miR-922 | -5.68 | 4.16E-03 |
| hsa-miR-664-3p | 13.51 | 7.52E-06 | hsa-miR-1251 | -5.64 | 3.13E-02 |
| hsa-miR-1287 | 13.50 | 1.35E-04 | hsa-miR-215 | -5.42 | 2.98E-02 |
| hsa-miR-130b-3p | 13.25 | 3.65E-04 | hsa-miR-526a | -5.36 | 1.82E-02 |
| hsa-miR-148a-3p | 12.97 | 9.55E-06 | hsa-miR-654-3p | -5.27 | 1.91E-02 |
| hsa-miR-651 | 12.90 | 5.56E-04 | hsa-miR-302d-3p | -5.22 | 2.68E-05 |
| hsa-miR-507 | 12.80 | 5.16E-03 | hsa-miR-614 | -5.19 | 3.38E-02 |
| hsa-miR-23b-3p | 12.70 | 7.35E-06 | hsa-miR-492 | -5.18 | 4.33E-02 |
| hsa-miR-573 | 12.64 | 2.13E-03 | hsa-miR-129-2-3p | -5.18 | 1.31E-02 |
| hsa-miR-3195 | 12.63 | 7.55E-05 | hsa-miR-369-5p | -5.17 | 2.88E-02 |
| hsa-miR-421 | 12.59 | 9.60E-05 | hsa-miR-502-5p | -5.09 | 2.19E-02 |
| hsa-miR-26a-5p | 12.36 | 5.76E-08 | hsa-miR-770-5p | -5.05 | 1.52E-02 |
| hsa-miR-4451 | 12.35 | 1.61E-04 | hsa-miR-1273d | -4.97 | 5.52E-03 |
| hsa-miR-508-3p | 12.27 | 2.19E-02 | hsa-miR-1185-5p | -4.96 | 1.89E-02 |
| hsa-miR-609 | 12.01 | 5.07E-04 | hsa-miR-1255b-5p | -4.94 | 3.71E-02 |
| hsa-miR-134 | 11.72 | 2.37E-03 | hsa-miR-516a-3p | -4.87 | 8.86E-03 |
| hsa-miR-27b-3p | 11.61 | 1.19E-05 | hsa-miR-892b | -4.82 | 2.66E-02 |
| hsa-miR-603 | 11.53 | 5.03E-03 | hsa-miR-548am-3p | -4.55 | 4.20E-03 |
| hsa-miR-299-3p | 10.86 | 1.37E-04 | hsa-miR-412 | -4.54 | 4.44E-02 |
| hsa-miR-483-5p | 10.76 | 2.79E-03 | hsa-miR-638 | -4.51 | 3.09E-02 |
| hsa-miR-4425 | 10.74 | 1.09E-03 | hsa-miR-659-3p | -4.39 | 4.76E-02 |
| hsa-miR-484 | 10.59 | 5.79E-07 | hsa-miR-513a-3p | -4.33 | 5.12E-03 |
| hsa-miR-4488 | 10.56 | 4.83E-04 | hsa-miR-627 | -4.32 | 1.82E-02 |
| hsa-miR-93-5p | 10.52 | 2.53E-11 | hsa-miR-767-3p | -4.28 | 2.52E-02 |
| hsa-miR-193a-3p | 10.44 | 5.85E-04 | hsa-miR-302b-3p | -4.25 | 3.52E-02 |
| hsa-miR-500b | 10.42 | 2.07E-03 | hsa-miR-635 | -4.23 | 4.30E-02 |
| hsa-miR-652-3p | 10.35 | 3.40E-03 | hsa-miR-154-5p | -4.15 | 3.67E-02 |
| hsa-miR-96-5p | 10.21 | 9.32E-05 | hsa-miR-1272 | -4.09 | 6.66E-03 |
| hsa-miR-567 | 10.18 | 2.92E-03 | hsa-miR-216a | -4.04 | 2.83E-02 |
| hsa-miR-145-5p | 10.12 | 1.30E-03 | hsa-miR-598 | -3.97 | 7.87E-03 |
| hsa-miR-23a-3p | 9.96 | 2.87E-07 | hsa-miR-3190-5p | -3.84 | 3.10E-03 |
| hsa-miR-106b-5p | 9.83 | 2.41E-09 | hsa-miR-587 | -3.72 | 3.17E-02 |
| hsa-miR-151a-5p | 9.75 | 3.18E-05 | hsa-miR-1245b-3p | -3.66 | 1.34E-02 |
| hsa-miR-361-5p | 9.74 | 2.24E-06 | hsa-miR-1267 | -3.56 | 4.97E-02 |
| hsa-miR-18a-5p | 9.69 | 3.42E-06 | hsa-miR-634 | -3.53 | 3.82E-02 |
| hsa-miR-3185 | 9.67 | 1.49E-03 | hsa-miR-320a | -3.46 | 1.30E-02 |
| hsa-miR-1225-5p | 9.64 | 8.80E-04 | hsa-miR-579 | -3.40 | 1.43E-02 |
| hsa-miR-92b-3p | 9.59 | 9.74E-03 | hsa-miR-2116-5p | -3.39 | 3.28E-02 |
| hsa-miR-1470 | 9.57 | 4.13E-03 | hsa-miR-548d-3p | -3.36 | 2.35E-03 |
| hsa-miR-342-3p | 9.55 | 1.99E-07 | hsa-miR-1286 | -3.33 | 4.84E-02 |
| hsa-miR-330-5p | 9.40 | 1.02E-03 | hsa-miR-612 | -3.30 | 1.03E-03 |
| hsa-miR-1206 | 9.31 | 6.24E-04 | hsa-miR-518b | -3.21 | 3.34E-02 |
| hsa-miR-604 | 9.05 | 5.93E-03 | hsa-miR-382-5p | -3.18 | 2.50E-02 |
| hsa-miR-373-3p | 9.00 | 1.26E-02 | hsa-miR-192-5p | -3.17 | 2.23E-02 |
| hsa-miR-3147 | 9.00 | 1.91E-03 | hsa-miR-139-3p | -3.17 | 2.64E-02 |
| hsa-miR-20a-5p | 8.92 | 2.30E-06 | hsa-miR-411-5p | -2.92 | 1.20E-02 |
| hsa-miR-520a-5p | 8.88 | 4.66E-03 | hsa-miR-548ai | -2.91 | 2.96E-02 |
| hsa-miR-1306-3p | 8.77 | 8.99E-03 | hsa-miR-302f | -2.75 | 3.19E-02 |
| hsa-miR-542-5p | 8.73 | 3.16E-03 | hsa-miR-485-3p | -2.66 | 3.90E-02 |
| hsa-miR-125a-3p | 8.63 | 1.54E-06 | hsa-miR-135b-5p | -2.60 | 4.49E-02 |
| hsa-miR-1321 | 8.44 | 3.16E-03 | hsa-miR-504 | -2.55 | 9.15E-03 |
| hsa-miR-875-5p | 8.37 | 4.87E-03 | hsa-miR-655 | -2.54 | 1.98E-02 |
| hsa-miR-26b-5p | 8.29 | 7.54E-07 | hsa-miR-337-3p | -2.40 | 3.07E-02 |
| hsa-miR-18b-5p | 8.26 | 1.47E-02 | hsa-miR-371a-3p | -2.40 | 4.43E-02 |
| hsa-miR-708-5p | 8.16 | 2.03E-03 | hsa-miR-495 | -2.37 | 3.31E-02 |
| hsa-miR-1913 | 7.99 | 4.57E-03 | hsa-miR-888-5p | -2.27 | 2.96E-02 |
| hsa-miR-1180 | 7.95 | 4.62E-04 |  |  |  |
| hsa-miR-30e-5p | 7.93 | 1.26E-06 |  |  |  |
| hsa-miR-521 | 7.91 | 8.98E-03 |  |  |  |
| hsa-miR-501-3p | 7.89 | 6.03E-03 |  |  |  |
| hsa-miR-505-3p | 7.85 | 1.95E-03 |  |  |  |
| hsa-miR-650 | 7.73 | 3.07E-03 |  |  |  |
| hsa-miR-331-3p | 7.69 | 2.15E-05 |  |  |  |
| hsa-miR-628-3p | 7.64 | 3.54E-03 |  |  |  |
| hsa-miR-518e-3p | 7.43 | 1.57E-02 |  |  |  |
| hsa-miR-296-5p | 7.23 | 7.01E-04 |  |  |  |
| hsa-miR-300 | 7.15 | 1.25E-02 |  |  |  |
| hsa-miR-941 | 7.14 | 2.95E-03 |  |  |  |
| hsa-miR-524-5p | 7.12 | 9.80E-03 |  |  |  |
| hsa-miR-151a-3p | 7.10 | 8.14E-05 |  |  |  |
| hsa-miR-155-5p | 7.00 | 4.69E-02 |  |  |  |
| hsa-miR-299-5p | 6.94 | 6.73E-03 |  |  |  |
| hsa-miR-182-5p | 6.93 | 1.84E-03 |  |  |  |
| hsa-miR-548y | 6.93 | 1.96E-02 |  |  |  |
| hsa-miR-496 | 6.66 | 6.82E-03 |  |  |  |
| hsa-miR-374b-5p | 6.66 | 2.83E-04 |  |  |  |
| hsa-miR-506-3p | 6.57 | 2.26E-02 |  |  |  |
| hsa-miR-371b-5p | 6.57 | 1.17E-02 |  |  |  |
| hsa-miR-339-5p | 6.57 | 1.94E-02 |  |  |  |
| hsa-miR-19b-3p | 6.53 | 1.48E-05 |  |  |  |
| hsa-miR-1275 | 6.51 | 4.95E-03 |  |  |  |
| hsa-miR-21-5p | 6.50 | 5.65E-04 |  |  |  |
| hsa-miR-208a | 6.45 | 1.66E-02 |  |  |  |
| hsa-miR-647 | 6.42 | 2.16E-02 |  |  |  |
| hsa-miR-548u | 6.30 | 3.09E-02 |  |  |  |
| hsa-miR-15b-5p | 6.28 | 3.13E-06 |  |  |  |
| hsa-miR-548n | 6.26 | 8.00E-03 |  |  |  |
| hsa-miR-628-5p | 6.16 | 1.17E-02 |  |  |  |
| hsa-miR-376c | 6.16 | 6.74E-03 |  |  |  |
| hsa-miR-649 | 6.16 | 3.37E-02 |  |  |  |
| hsa-miR-4792 | 6.13 | 2.10E-02 |  |  |  |
| hsa-miR-616-3p | 6.05 | 1.13E-02 |  |  |  |
| hsa-miR-483-3p | 6.02 | 5.07E-03 |  |  |  |
| hsa-miR-101-3p | 5.95 | 5.29E-03 |  |  |  |
| hsa-miR-582-5p | 5.92 | 1.06E-02 |  |  |  |
| hsa-miR-371b-3p | 5.91 | 1.75E-02 |  |  |  |
| hsa-miR-185-5p | 5.89 | 1.26E-04 |  |  |  |
| hsa-miR-2114-5p | 5.82 | 2.62E-02 |  |  |  |
| hsa-miR-620 | 5.81 | 2.54E-02 |  |  |  |
| hsa-miR-921 | 5.77 | 2.33E-02 |  |  |  |
| hsa-miR-588 | 5.76 | 3.46E-02 |  |  |  |
| hsa-miR-924 | 5.74 | 2.75E-02 |  |  |  |
| hsa-miR-874 | 5.66 | 3.18E-02 |  |  |  |
| hsa-miR-1914-5p | 5.65 | 3.57E-02 |  |  |  |
| hsa-miR-4532 | 5.65 | 1.14E-02 |  |  |  |
| hsa-miR-1910 | 5.53 | 4.07E-02 |  |  |  |
| hsa-miR-548a-3p | 5.37 | 3.57E-02 |  |  |  |
| hsa-miR-3154 | 5.32 | 4.46E-02 |  |  |  |
| hsa-miR-517c-3p | 5.22 | 1.41E-02 |  |  |  |
| hsa-miR-422a | 5.21 | 3.51E-02 |  |  |  |
| hsa-miR-590-3p | 5.17 | 1.48E-02 |  |  |  |
| hsa-miR-320d | 5.15 | 1.47E-02 |  |  |  |
| hsa-miR-597 | 5.10 | 1.61E-02 |  |  |  |
| hsa-miR-320b | 5.07 | 3.78E-02 |  |  |  |
| hsa-miR-455-5p | 4.97 | 1.06E-02 |  |  |  |
| hsa-miR-1261 | 4.92 | 2.50E-02 |  |  |  |
| hsa-miR-593-3p | 4.86 | 3.85E-02 |  |  |  |
| hsa-miR-3690 | 4.85 | 9.15E-03 |  |  |  |
| hsa-miR-1972 | 4.79 | 4.30E-02 |  |  |  |
| hsa-miR-519d | 4.75 | 3.57E-03 |  |  |  |
| hsa-miR-423-3p | 4.74 | 3.77E-03 |  |  |  |
| hsa-miR-744-5p | 4.73 | 3.95E-02 |  |  |  |
| hsa-miR-1273e | 4.71 | 4.26E-02 |  |  |  |
| hsa-miR-193b-3p | 4.69 | 1.02E-02 |  |  |  |
| hsa-miR-376b | 4.66 | 1.15E-02 |  |  |  |
| hsa-miR-1265 | 4.65 | 4.75E-02 |  |  |  |
| hsa-miR-181b-5p | 4.64 | 7.54E-05 |  |  |  |
| hsa-miR-197-3p | 4.58 | 3.23E-03 |  |  |  |
| hsa-miR-449a | 4.56 | 2.43E-02 |  |  |  |
| hsa-miR-944 | 4.55 | 4.81E-02 |  |  |  |
| hsa-miR-639 | 4.49 | 4.68E-02 |  |  |  |
| hsa-miR-1307-3p | 4.47 | 4.55E-02 |  |  |  |
| hsa-miR-875-3p | 4.42 | 3.32E-02 |  |  |  |
| hsa-miR-146a-5p | 4.39 | 2.18E-02 |  |  |  |
| hsa-miR-103a-3p | 4.37 | 4.65E-02 |  |  |  |
| hsa-miR-301a-3p | 4.35 | 5.06E-04 |  |  |  |
| hsa-miR-548t-5p | 4.31 | 3.68E-02 |  |  |  |
| hsa-miR-1468 | 4.23 | 4.83E-02 |  |  |  |
| hsa-miR-4454 | 4.21 | 2.16E-02 |  |  |  |
| hsa-miR-624-3p | 4.20 | 4.00E-02 |  |  |  |
| hsa-miR-497-5p | 4.18 | 4.37E-02 |  |  |  |
| hsa-miR-574-3p | 4.03 | 3.62E-03 |  |  |  |
| hsa-miR-448 | 4.02 | 3.89E-02 |  |  |  |
| hsa-miR-15a-5p | 3.95 | 3.39E-05 |  |  |  |
| hsa-miR-640 | 3.82 | 3.62E-02 |  |  |  |
| hsa-miR-663a | 3.74 | 4.67E-02 |  |  |  |
| hsa-let-7g-5p | 3.70 | 8.58E-05 |  |  |  |
| hsa-miR-130a-3p | 3.62 | 1.55E-03 |  |  |  |
| hsa-miR-186-5p | 3.61 | 2.88E-02 |  |  |  |
| hsa-miR-28-5p | 3.52 | 1.43E-02 |  |  |  |
| hsa-miR-409-3p | 3.46 | 4.04E-02 |  |  |  |
| hsa-miR-107 | 3.45 | 7.67E-03 |  |  |  |
| hsa-miR-16-5p | 3.33 | 6.13E-03 |  |  |  |
| hsa-miR-210 | 3.31 | 3.69E-03 |  |  |  |
| hsa-miR-374a-5p | 3.25 | 1.42E-03 |  |  |  |
| hsa-miR-548c-5p | 3.15 | 4.06E-02 |  |  |  |
| hsa-let-7b-5p | 3.05 | 2.96E-03 |  |  |  |
| hsa-miR-891a | 2.94 | 4.16E-02 |  |  |  |
| hsa-let-7a-5p | 2.93 | 3.26E-03 |  |  |  |
| hsa-miR-200b-3p | 2.73 | 3.77E-02 |  |  |  |
| hsa-let-7c | 2.71 | 4.45E-02 |  |  |  |
| hsa-miR-1202 | 2.69 | 2.10E-02 |  |  |  |
| hsa-miR-19a-3p | 2.68 | 3.96E-02 |  |  |  |
| hsa-miR-30a-5p | 2.65 | 3.11E-02 |  |  |  |
| hsa-miR-181a-5p | 2.61 | 2.54E-02 |  |  |  |
| hsa-miR-32-5p | 2.36 | 4.70E-02 |  |  |  |
| hsa-miR-25-3p | 2.13 | 1.10E-02 |  |  |  |
| hsa-miR-191-5p | 2.07 | 3.87E-02 |  |  |  |

*compared to normal fallopian tube.
