# Supplementary material for: Identification of core aberrantly expressed microRNAs in serous ovarian carcinoma
Source: Oncotarget. 2018 Apr 17;9(29):20451–66. doi: 10.18632/oncotarget.24942 (PMC5945511; doi:10.18632/oncotarget.24942)
Supplement: Supplementary file 4 [file oncotarget-09-20451-s004.docx]

**Supplementary Table 5.** Aberrantly expressed miRNAs in primary resistant/refractory tumors

| Up-regulated | | | | Down-regulated | | |
| --- | --- | --- | --- | --- | --- | --- |
| Name | Fold Change* | | *P*-value | Name | Fold Change* | *P*-value |
| hsa-miR-143-3p | 447.34 | 1.48E-09 | | hsa-miR-3182 | -9.71 | 9.77E-04 |
| hsa-miR-203 | 331.38 | 1.10E-17 | | hsa-miR-938 | -8.75 | 7.33E-03 |
| hsa-miR-199a-3p | 274.37 | 4.56E-12 | | hsa-miR-486-3p | -8.61 | 5.79E-03 |
| hsa-miR-199b-5p | 244.71 | 1.64E-11 | | hsa-miR-767-5p | -8.60 | 1.83E-02 |
| hsa-miR-150-5p | 173.85 | 9.56E-09 | | hsa-miR-3136-5p | -8.54 | 3.11E-03 |
| hsa-miR-205-5p | 159.53 | 2.70E-08 | | hsa-miR-548d-5p | -7.74 | 4.55E-03 |
| hsa-miR-4286 | 135.54 | 2.59E-10 | | hsa-miR-646 | -7.63 | 1.02E-02 |
| hsa-miR-451a | 129.19 | 1.13E-08 | | hsa-miR-515-5p | -7.31 | 7.46E-03 |
| hsa-miR-223-3p | 116.77 | 7.86E-12 | | hsa-miR-550a-5p | -7.25 | 1.21E-02 |
| hsa-miR-425-5p | 92.35 | 3.75E-16 | | hsa-miR-576-3p | -6.34 | 1.76E-02 |
| hsa-miR-500a-5p | 85.26 | 2.61E-11 | | hsa-miR-518a-3p | -6.04 | 1.12E-02 |
| hsa-miR-429 | 84.48 | 8.15E-15 | | hsa-miR-1288 | -5.61 | 2.80E-02 |
| hsa-miR-30c-5p | 78.16 | 1.26E-11 | | hsa-miR-450b-3p | -5.51 | 9.00E-03 |
| hsa-miR-1246 | 76.66 | 2.66E-03 | | hsa-miR-576-5p | -5.48 | 3.95E-02 |
| hsa-miR-1260b | 76.07 | 9.97E-12 | | hsa-miR-520c-3p | -5.27 | 2.49E-02 |
| hsa-miR-575 | 61.78 | 3.24E-09 | | hsa-miR-548an | -5.18 | 7.98E-03 |
| hsa-miR-181c-5p | 58.45 | 9.32E-09 | | hsa-miR-580 | -5.18 | 2.32E-02 |
| hsa-miR-423-5p | 54.41 | 2.14E-12 | | hsa-miR-3605-5p | -4.99 | 1.56E-02 |
| hsa-miR-27a-3p | 53.54 | 1.29E-10 | | hsa-miR-562 | -4.99 | 3.89E-02 |
| hsa-miR-454-3p | 42.56 | 2.36E-07 | | hsa-miR-499a-3p | -4.97 | 2.01E-02 |
| hsa-miR-345-5p | 42.17 | 3.89E-10 | | hsa-miR-761 | -4.75 | 7.00E-03 |
| hsa-miR-10b-5p | 40.23 | 6.60E-11 | | hsa-miR-1284 | -4.69 | 1.51E-02 |
| hsa-miR-99a-5p | 36.36 | 9.66E-09 | | hsa-miR-1324 | -4.63 | 5.55E-03 |
| hsa-miR-340-5p | 36.09 | 9.01E-12 | | hsa-miR-2277-3p | -4.57 | 2.95E-02 |
| hsa-miR-720 | 34.38 | 1.71E-08 | | hsa-miR-762 | -4.54 | 2.60E-02 |
| hsa-miR-106a-5p | 33.58 | 1.29E-16 | | hsa-miR-548ak | -4.52 | 4.19E-02 |
| hsa-miR-1915-3p | 32.68 | 1.39E-06 | | hsa-miR-1205 | -4.49 | 4.75E-02 |
| hsa-miR-30d-5p | 32.53 | 5.37E-09 | | hsa-miR-1273d | -4.42 | 9.05E-03 |
| hsa-miR-142-3p | 31.44 | 5.47E-08 | | hsa-miR-4458 | -4.40 | 4.27E-03 |
| hsa-miR-146b-5p | 30.58 | 4.22E-07 | | hsa-miR-1183 | -4.40 | 4.48E-02 |
| hsa-miR-221-3p | 30.49 | 8.65E-10 | | hsa-miR-485-5p | -4.32 | 3.75E-02 |
| hsa-miR-1287 | 30.07 | 9.51E-07 | | hsa-miR-3168 | -4.30 | 7.98E-03 |
| hsa-miR-660-5p | 30.01 | 1.44E-07 | | hsa-miR-302d-3p | -4.21 | 1.83E-04 |
| hsa-miR-1301 | 29.67 | 1.91E-06 | | hsa-miR-302e | -4.10 | 4.04E-02 |
| hsa-miR-320e | 29.43 | 2.61E-08 | | hsa-miR-367-3p | -4.10 | 2.31E-02 |
| hsa-miR-1260a | 29.31 | 4.36E-11 | | hsa-miR-1 | -4.09 | 2.89E-02 |
| hsa-miR-617 | 28.99 | 2.33E-05 | | hsa-miR-339-3p | -4.08 | 4.04E-02 |
| hsa-miR-199a-5p | 28.93 | 3.65E-07 | | hsa-miR-1245b-5p | -4.06 | 3.19E-02 |
| hsa-miR-596 | 27.70 | 9.48E-07 | | hsa-miR-3175 | -4.00 | 1.52E-02 |
| hsa-miR-92a-3p | 27.70 | 7.54E-10 | | hsa-miR-1272 | -3.99 | 6.97E-03 |
| hsa-miR-455-3p | 27.59 | 2.81E-05 | | hsa-miR-3190-5p | -3.85 | 2.73E-03 |
| hsa-miR-200c-3p | 26.95 | 6.51E-10 | | hsa-miR-625-5p | -3.82 | 3.85E-02 |
| hsa-miR-214-3p | 26.70 | 3.08E-06 | | hsa-miR-1257 | -3.77 | 3.87E-02 |
| hsa-miR-183-5p | 26.35 | 4.41E-09 | | hsa-miR-767-3p | -3.67 | 4.23E-02 |
| hsa-miR-609 | 24.96 | 8.79E-06 | | hsa-miR-548am-3p | -3.65 | 1.26E-02 |
| hsa-miR-126-3p | 23.41 | 6.11E-12 | | hsa-miR-1908 | -3.65 | 1.73E-02 |
| hsa-miR-542-5p | 23.06 | 2.60E-05 | | hsa-miR-3187-3p | -3.59 | 3.78E-02 |
| hsa-miR-299-3p | 22.71 | 9.46E-07 | | hsa-miR-655 | -3.42 | 2.19E-03 |
| hsa-miR-362-5p | 22.71 | 2.86E-07 | | hsa-miR-922 | -3.38 | 3.94E-02 |
| hsa-miR-421 | 22.28 | 2.27E-06 | | hsa-miR-598 | -3.29 | 1.95E-02 |
| hsa-miR-130b-3p | 22.26 | 2.07E-05 | | hsa-miR-371a-3p | -3.29 | 6.18E-03 |
| hsa-miR-187-3p | 21.07 | 1.53E-06 | | hsa-miR-631 | -3.11 | 1.27E-02 |
| hsa-miR-30b-5p | 20.26 | 8.91E-08 | | hsa-miR-1825 | -3.08 | 1.15E-02 |
| hsa-miR-664-3p | 19.96 | 3.20E-07 | | hsa-miR-548d-3p | -2.98 | 5.32E-03 |
| hsa-miR-193a-3p | 19.51 | 1.61E-05 | | hsa-miR-188-5p | -2.89 | 3.13E-02 |
| hsa-miR-4425 | 19.01 | 5.60E-05 | | hsa-miR-513a-3p | -2.84 | 4.12E-02 |
| hsa-miR-194-5p | 18.61 | 4.58E-06 | | hsa-miR-504 | -2.67 | 5.91E-03 |
| hsa-miR-141-3p | 18.57 | 1.68E-09 | | hsa-miR-612 | -2.55 | 8.38E-03 |
| hsa-miR-148a-3p | 18.55 | 5.27E-07 | | hsa-miR-888-5p | -2.19 | 3.44E-02 |
| hsa-miR-651 | 17.49 | 1.08E-04 | |  |  |  |
| hsa-miR-24-3p | 17.13 | 1.52E-08 | |  |  |  |
| hsa-miR-324-5p | 17.09 | 2.57E-08 | |  |  |  |
| hsa-miR-3195 | 16.96 | 1.00E-05 | |  |  |  |
| hsa-miR-514a-3p | 16.63 | 4.99E-03 | |  |  |  |
| hsa-miR-4488 | 16.41 | 3.66E-05 | |  |  |  |
| hsa-miR-200a-3p | 16.32 | 4.35E-09 | |  |  |  |
| hsa-miR-1225-5p | 16.16 | 4.83E-05 | |  |  |  |
| hsa-miR-155-5p | 15.74 | 4.90E-03 | |  |  |  |
| hsa-miR-483-5p | 15.63 | 5.25E-04 | |  |  |  |
| hsa-miR-520h | 15.58 | 4.15E-05 | |  |  |  |
| hsa-miR-1206 | 15.53 | 2.93E-05 | |  |  |  |
| hsa-miR-4451 | 15.32 | 3.93E-05 | |  |  |  |
| hsa-miR-134 | 15.13 | 7.46E-04 | |  |  |  |
| hsa-miR-652-3p | 15.12 | 6.44E-04 | |  |  |  |
| hsa-miR-708-5p | 15.09 | 7.52E-05 | |  |  |  |
| hsa-miR-26a-5p | 14.93 | 5.94E-09 | |  |  |  |
| hsa-miR-23b-3p | 14.69 | 1.95E-06 | |  |  |  |
| hsa-miR-27b-3p | 14.66 | 1.63E-06 | |  |  |  |
| hsa-miR-4516 | 14.01 | 2.19E-06 | |  |  |  |
| hsa-miR-3147 | 13.74 | 2.16E-04 | |  |  |  |
| hsa-miR-145-5p | 13.57 | 2.80E-04 | |  |  |  |
| hsa-miR-96-5p | 13.43 | 1.25E-05 | |  |  |  |
| hsa-miR-484 | 13.43 | 4.32E-08 | |  |  |  |
| hsa-miR-299-5p | 13.34 | 3.15E-04 | |  |  |  |
| hsa-miR-18b-5p | 13.31 | 2.72E-03 | |  |  |  |
| hsa-miR-378d | 13.20 | 1.90E-03 | |  |  |  |
| hsa-miR-941 | 13.12 | 1.11E-04 | |  |  |  |
| hsa-miR-362-3p | 12.83 | 1.03E-04 | |  |  |  |
| hsa-miR-361-5p | 12.67 | 1.51E-07 | |  |  |  |
| hsa-miR-93-5p | 12.47 | 1.27E-12 | |  |  |  |
| hsa-miR-23a-3p | 12.43 | 2.11E-08 | |  |  |  |
| hsa-miR-208a | 12.33 | 1.28E-03 | |  |  |  |
| hsa-miR-330-5p | 12.29 | 2.23E-04 | |  |  |  |
| hsa-miR-20a-5p | 12.27 | 8.07E-08 | |  |  |  |
| hsa-miR-573 | 12.09 | 2.25E-03 | |  |  |  |
| hsa-miR-513b | 12.08 | 3.11E-02 | |  |  |  |
| hsa-miR-151a-5p | 11.85 | 6.02E-06 | |  |  |  |
| hsa-miR-342-3p | 11.69 | 1.64E-08 | |  |  |  |
| hsa-miR-92b-3p | 11.43 | 4.98E-03 | |  |  |  |
| hsa-miR-513c-5p | 11.29 | 1.18E-02 | |  |  |  |
| hsa-miR-1275 | 11.29 | 2.94E-04 | |  |  |  |
| hsa-miR-520a-5p | 11.23 | 1.62E-03 | |  |  |  |
| hsa-miR-532-3p | 11.18 | 7.47E-04 | |  |  |  |
| hsa-miR-106b-5p | 11.11 | 3.38E-10 | |  |  |  |
| hsa-miR-376c | 11.04 | 3.68E-04 | |  |  |  |
| hsa-miR-483-3p | 10.94 | 2.06E-04 | |  |  |  |
| hsa-miR-320d | 10.89 | 4.19E-04 | |  |  |  |
| hsa-miR-500b | 10.79 | 1.59E-03 | |  |  |  |
| hsa-miR-1470 | 10.74 | 2.36E-03 | |  |  |  |
| hsa-miR-125a-3p | 10.41 | 1.81E-07 | |  |  |  |
| hsa-miR-1180 | 10.22 | 8.44E-05 | |  |  |  |
| hsa-miR-521 | 10.16 | 3.21E-03 | |  |  |  |
| hsa-miR-18a-5p | 10.02 | 2.02E-06 | |  |  |  |
| hsa-miR-182-5p | 9.97 | 2.18E-04 | |  |  |  |
| hsa-miR-875-5p | 9.84 | 2.26E-03 | |  |  |  |
| hsa-miR-26b-5p | 9.83 | 9.33E-08 | |  |  |  |
| hsa-miR-628-5p | 9.81 | 1.55E-03 | |  |  |  |
| hsa-miR-1913 | 9.70 | 1.80E-03 | |  |  |  |
| hsa-miR-517c-3p | 9.51 | 8.66E-04 | |  |  |  |
| hsa-miR-567 | 9.47 | 3.49E-03 | |  |  |  |
| hsa-miR-603 | 9.36 | 9.20E-03 | |  |  |  |
| hsa-miR-30e-5p | 9.31 | 1.78E-07 | |  |  |  |
| hsa-miR-548n | 9.26 | 1.27E-03 | |  |  |  |
| hsa-miR-496 | 9.12 | 1.55E-03 | |  |  |  |
| hsa-miR-501-3p | 9.11 | 3.07E-03 | |  |  |  |
| hsa-miR-944 | 9.07 | 4.07E-03 | |  |  |  |
| hsa-miR-21-5p | 9.02 | 5.30E-05 | |  |  |  |
| hsa-miR-4532 | 8.92 | 1.38E-03 | |  |  |  |
| hsa-miR-1306-3p | 8.73 | 8.38E-03 | |  |  |  |
| hsa-miR-604 | 8.68 | 6.30E-03 | |  |  |  |
| hsa-miR-376b | 8.67 | 4.29E-04 | |  |  |  |
| hsa-miR-1261 | 8.66 | 2.41E-03 | |  |  |  |
| hsa-miR-331-3p | 8.62 | 6.53E-06 | |  |  |  |
| hsa-miR-647 | 8.57 | 7.59E-03 | |  |  |  |
| hsa-miR-505-3p | 8.49 | 1.17E-03 | |  |  |  |
| hsa-miR-582-5p | 8.31 | 2.27E-03 | |  |  |  |
| hsa-miR-3185 | 8.31 | 2.62E-03 | |  |  |  |
| hsa-miR-524-5p | 8.25 | 5.12E-03 | |  |  |  |
| hsa-miR-146a-5p | 8.23 | 1.15E-03 | |  |  |  |
| hsa-miR-455-5p | 8.22 | 8.19E-04 | |  |  |  |
| hsa-miR-374b-5p | 8.08 | 6.01E-05 | |  |  |  |
| hsa-miR-339-5p | 8.05 | 9.04E-03 | |  |  |  |
| hsa-miR-206 | 8.03 | 6.79E-03 | |  |  |  |
| hsa-miR-296-5p | 8.01 | 3.28E-04 | |  |  |  |
| hsa-miR-3690 | 7.96 | 6.47E-04 | |  |  |  |
| hsa-miR-1178 | 7.90 | 1.30E-02 | |  |  |  |
| hsa-miR-588 | 7.84 | 1.24E-02 | |  |  |  |
| hsa-miR-1468 | 7.79 | 4.96E-03 | |  |  |  |
| hsa-miR-593-3p | 7.63 | 7.63E-03 | |  |  |  |
| hsa-miR-2114-5p | 7.62 | 9.89E-03 | |  |  |  |
| hsa-miR-525-3p | 7.61 | 6.52E-03 | |  |  |  |
| hsa-miR-597 | 7.45 | 2.96E-03 | |  |  |  |
| hsa-miR-373-3p | 7.42 | 2.09E-02 | |  |  |  |
| hsa-miR-151a-3p | 7.37 | 5.06E-05 | |  |  |  |
| hsa-miR-924 | 7.31 | 1.15E-02 | |  |  |  |
| hsa-miR-101-3p | 7.28 | 1.81E-03 | |  |  |  |
| hsa-miR-1321 | 7.25 | 5.38E-03 | |  |  |  |
| hsa-miR-639 | 7.24 | 8.60E-03 | |  |  |  |
| hsa-miR-509-3-5p | 7.18 | 2.68E-02 | |  |  |  |
| hsa-miR-1292 | 7.11 | 1.71E-02 | |  |  |  |
| hsa-miR-650 | 7.08 | 4.09E-03 | |  |  |  |
| hsa-miR-423-3p | 7.06 | 2.84E-04 | |  |  |  |
| hsa-miR-497-5p | 7.03 | 5.93E-03 | |  |  |  |
| hsa-miR-193b-3p | 7.01 | 1.21E-03 | |  |  |  |
| hsa-miR-548t-5p | 6.87 | 5.80E-03 | |  |  |  |
| hsa-miR-1972 | 6.74 | 1.32E-02 | |  |  |  |
| hsa-miR-590-3p | 6.59 | 4.91E-03 | |  |  |  |
| hsa-miR-185-5p | 6.56 | 4.35E-05 | |  |  |  |
| hsa-miR-548a-3p | 6.44 | 1.89E-02 | |  |  |  |
| hsa-miR-320b | 6.44 | 1.64E-02 | |  |  |  |
| hsa-miR-300 | 6.37 | 1.72E-02 | |  |  |  |
| hsa-miR-518e-3p | 6.35 | 2.39E-02 | |  |  |  |
| hsa-miR-19b-3p | 6.31 | 1.67E-05 | |  |  |  |
| hsa-miR-422a | 6.27 | 1.81E-02 | |  |  |  |
| hsa-miR-224-5p | 6.25 | 1.58E-02 | |  |  |  |
| hsa-miR-1265 | 6.22 | 1.77E-02 | |  |  |  |
| hsa-miR-136-5p | 6.17 | 1.10E-02 | |  |  |  |
| hsa-miR-15b-5p | 6.15 | 3.10E-06 | |  |  |  |
| hsa-miR-302a-3p | 6.15 | 8.00E-03 | |  |  |  |
| hsa-miR-1307-3p | 6.05 | 1.56E-02 | |  |  |  |
| hsa-miR-548w | 6.01 | 2.55E-02 | |  |  |  |
| hsa-miR-186-5p | 5.95 | 2.44E-03 | |  |  |  |
| hsa-miR-383 | 5.92 | 9.42E-03 | |  |  |  |
| hsa-miR-1280 | 5.88 | 1.18E-02 | |  |  |  |
| hsa-miR-146b-3p | 5.87 | 1.59E-02 | |  |  |  |
| hsa-miR-3154 | 5.84 | 3.22E-02 | |  |  |  |
| hsa-miR-433 | 5.83 | 2.22E-02 | |  |  |  |
| hsa-miR-202-3p | 5.75 | 1.31E-02 | |  |  |  |
| hsa-miR-629-5p | 5.71 | 1.90E-02 | |  |  |  |
| hsa-miR-675-5p | 5.68 | 4.39E-02 | |  |  |  |
| hsa-miR-624-3p | 5.67 | 1.25E-02 | |  |  |  |
| hsa-miR-331-5p | 5.64 | 8.93E-03 | |  |  |  |
| hsa-miR-301a-3p | 5.64 | 4.48E-05 | |  |  |  |
| hsa-miR-1303 | 5.53 | 1.91E-02 | |  |  |  |
| hsa-miR-1273e | 5.48 | 2.47E-02 | |  |  |  |
| hsa-miR-4454 | 5.48 | 6.31E-03 | |  |  |  |
| hsa-miR-548u | 5.46 | 4.35E-02 | |  |  |  |
| hsa-miR-3161 | 5.45 | 2.20E-02 | |  |  |  |
| hsa-miR-409-3p | 5.42 | 5.23E-03 | |  |  |  |
| hsa-miR-28-5p | 5.37 | 1.11E-03 | |  |  |  |
| hsa-miR-877-5p | 5.36 | 3.27E-02 | |  |  |  |
| hsa-miR-324-3p | 5.33 | 3.32E-02 | |  |  |  |
| hsa-miR-874 | 5.25 | 3.76E-02 | |  |  |  |
| hsa-miR-323a-5p | 5.24 | 3.33E-02 | |  |  |  |
| hsa-miR-181b-5p | 5.23 | 1.87E-05 | |  |  |  |
| hsa-miR-630 | 5.21 | 2.27E-02 | |  |  |  |
| hsa-miR-498 | 5.13 | 2.66E-02 | |  |  |  |
| hsa-miR-371b-3p | 5.11 | 2.70E-02 | |  |  |  |
| hsa-miR-548y | 5.07 | 4.65E-02 | |  |  |  |
| hsa-miR-2278 | 5.06 | 1.74E-02 | |  |  |  |
| hsa-miR-1193 | 4.98 | 2.56E-02 | |  |  |  |
| hsa-miR-875-3p | 4.98 | 2.02E-02 | |  |  |  |
| hsa-miR-600 | 4.98 | 2.87E-02 | |  |  |  |
| hsa-miR-376a-3p | 4.91 | 8.21E-03 | |  |  |  |
| hsa-miR-1262 | 4.83 | 2.89E-02 | |  |  |  |
| hsa-miR-663a | 4.81 | 1.71E-02 | |  |  |  |
| hsa-miR-301b | 4.79 | 1.11E-02 | |  |  |  |
| hsa-let-7c | 4.78 | 1.72E-03 | |  |  |  |
| hsa-miR-449a | 4.71 | 2.00E-02 | |  |  |  |
| hsa-miR-574-3p | 4.63 | 1.30E-03 | |  |  |  |
| hsa-miR-190b | 4.61 | 4.50E-02 | |  |  |  |
| hsa-miR-643 | 4.60 | 4.66E-02 | |  |  |  |
| hsa-miR-551b-3p | 4.60 | 4.60E-02 | |  |  |  |
| hsa-miR-744-5p | 4.59 | 4.09E-02 | |  |  |  |
| hsa-miR-640 | 4.53 | 1.75E-02 | |  |  |  |
| hsa-miR-448 | 4.52 | 2.37E-02 | |  |  |  |
| hsa-miR-130a-3p | 4.49 | 2.22E-04 | |  |  |  |
| hsa-miR-371b-5p | 4.47 | 4.10E-02 | |  |  |  |
| hsa-let-7g-5p | 4.42 | 8.49E-06 | |  |  |  |
| hsa-miR-577 | 4.41 | 4.19E-02 | |  |  |  |
| hsa-miR-3180-3p | 4.36 | 4.30E-02 | |  |  |  |
| hsa-miR-1297 | 4.27 | 4.32E-02 | |  |  |  |
| hsa-miR-519d | 4.26 | 5.97E-03 | |  |  |  |
| hsa-miR-592 | 4.18 | 2.08E-02 | |  |  |  |
| hsa-miR-329 | 4.16 | 4.54E-02 | |  |  |  |
| hsa-miR-2053 | 4.14 | 2.76E-02 | |  |  |  |
| hsa-miR-107 | 4.14 | 2.12E-03 | |  |  |  |
| hsa-miR-1252 | 4.13 | 2.92E-02 | |  |  |  |
| hsa-miR-181a-5p | 4.11 | 1.07E-03 | |  |  |  |
| hsa-miR-641 | 4.10 | 2.31E-02 | |  |  |  |
| hsa-miR-200b-3p | 4.10 | 3.54E-03 | |  |  |  |
| hsa-let-7b-5p | 4.08 | 1.89E-04 | |  |  |  |
| hsa-miR-381 | 4.00 | 3.84E-02 | |  |  |  |
| hsa-miR-16-5p | 3.97 | 1.61E-03 | |  |  |  |
| hsa-miR-15a-5p | 3.91 | 3.11E-05 | |  |  |  |
| hsa-let-7a-5p | 3.83 | 2.45E-04 | |  |  |  |
| hsa-miR-210 | 3.80 | 1.14E-03 | |  |  |  |
| hsa-miR-548m | 3.76 | 3.85E-02 | |  |  |  |
| hsa-miR-891a | 3.60 | 1.49E-02 | |  |  |  |
| hsa-miR-30a-5p | 3.58 | 4.75E-03 | |  |  |  |
| hsa-miR-19a-3p | 3.58 | 7.55E-03 | |  |  |  |
| hsa-miR-195-5p | 3.49 | 3.79E-02 | |  |  |  |
| hsa-miR-374a-5p | 3.48 | 6.89E-04 | |  |  |  |
| hsa-let-7e-5p | 3.38 | 3.45E-03 | |  |  |  |
| hsa-miR-197-3p | 3.36 | 1.67E-02 | |  |  |  |
| hsa-miR-379-5p | 3.12 | 4.25E-02 | |  |  |  |
| hsa-miR-125b-5p | 2.93 | 2.35E-02 | |  |  |  |
| hsa-miR-25-3p | 2.79 | 6.06E-04 | |  |  |  |
| hsa-miR-193a-5p | 2.76 | 2.68E-02 | |  |  |  |
| hsa-miR-98 | 2.72 | 1.76E-02 | |  |  |  |
| hsa-miR-1202 | 2.64 | 2.19E-02 | |  |  |  |
| hsa-miR-191-5p | 2.59 | 6.59E-03 | |  |  |  |
| hsa-miR-548aa | 2.36 | 3.64E-02 | |  |  |  |
| hsa-miR-32-5p | 2.34 | 4.69E-02 | |  |  |  |

*compared to normal fallopian tube.
