# Supplementary material for: Identification of core aberrantly expressed microRNAs in serous ovarian carcinoma
Source: Oncotarget. 2018 Apr 17;9(29):20451–66. doi: 10.18632/oncotarget.24942 (PMC5945511; doi:10.18632/oncotarget.24942)
Supplement: Supplementary file 5 [file oncotarget-09-20451-s005.docx]

**Supplementary Table 6.** Aberrantly expressed miRNAs only in primary resistant/refractory tumors, but not in primary chemo-sensitive tumors

| Up-regulated | | | Down-regulated | | |
| --- | --- | --- | --- | --- | --- |
| Name | Fold Change* | *P*-value | Name | Fold Change* | *P*-value |
| hsa-miR-206 | 8.03 | 6.79E-03 | hsa-miR-631 | -3.11 | 1.27E-02 |
| hsa-miR-1178 | 7.90 | 1.30E-02 | hsa-miR-188-5p | -2.89 | 3.13E-02 |
| hsa-miR-525-3p | 7.61 | 6.52E-03 |  |  |  |
| hsa-miR-1292 | 7.11 | 1.71E-02 |  |  |  |
| hsa-miR-224-5p | 6.25 | 1.58E-02 |  |  |  |
| hsa-miR-136-5p | 6.17 | 1.10E-02 |  |  |  |
| hsa-miR-302a-3p | 6.15 | 8.00E-03 |  |  |  |
| hsa-miR-548w | 6.01 | 2.55E-02 |  |  |  |
| hsa-miR-383 | 5.92 | 9.42E-03 |  |  |  |
| hsa-miR-1280 | 5.88 | 1.18E-02 |  |  |  |
| hsa-miR-146b-3p | 5.87 | 1.59E-02 |  |  |  |
| hsa-miR-433 | 5.83 | 2.22E-02 |  |  |  |
| hsa-miR-202-3p | 5.75 | 1.31E-02 |  |  |  |
| hsa-miR-629-5p | 5.71 | 1.90E-02 |  |  |  |
| hsa-miR-675-5p | 5.68 | 4.39E-02 |  |  |  |
| hsa-miR-331-5p | 5.64 | 8.93E-03 |  |  |  |
| hsa-miR-1303 | 5.53 | 1.91E-02 |  |  |  |
| hsa-miR-3161 | 5.45 | 2.20E-02 |  |  |  |
| hsa-miR-877-5p | 5.36 | 3.27E-02 |  |  |  |
| hsa-miR-324-3p | 5.33 | 3.32E-02 |  |  |  |
| hsa-miR-323a-5p | 5.24 | 3.33E-02 |  |  |  |
| hsa-miR-630 | 5.21 | 2.27E-02 |  |  |  |
| hsa-miR-498 | 5.13 | 2.66E-02 |  |  |  |
| hsa-miR-2278 | 5.06 | 1.74E-02 |  |  |  |
| hsa-miR-1193 | 4.98 | 2.56E-02 |  |  |  |
| hsa-miR-600 | 4.98 | 2.87E-02 |  |  |  |
| hsa-miR-376a-3p | 4.91 | 8.21E-03 |  |  |  |
| hsa-miR-1262 | 4.83 | 2.89E-02 |  |  |  |
| hsa-miR-301b | 4.79 | 1.11E-02 |  |  |  |
| hsa-miR-190b | 4.61 | 4.50E-02 |  |  |  |
| hsa-miR-643 | 4.60 | 4.66E-02 |  |  |  |
| hsa-miR-551b-3p | 4.60 | 4.60E-02 |  |  |  |
| hsa-miR-577 | 4.41 | 4.19E-02 |  |  |  |
| hsa-miR-3180-3p | 4.36 | 4.30E-02 |  |  |  |
| hsa-miR-1297 | 4.27 | 4.32E-02 |  |  |  |
| hsa-miR-592 | 4.18 | 2.08E-02 |  |  |  |
| hsa-miR-329 | 4.16 | 4.54E-02 |  |  |  |
| hsa-miR-2053 | 4.14 | 2.76E-02 |  |  |  |
| hsa-miR-1252 | 4.13 | 2.92E-02 |  |  |  |
| hsa-miR-641 | 4.10 | 2.31E-02 |  |  |  |
| hsa-miR-381 | 4.00 | 3.84E-02 |  |  |  |
| hsa-miR-548m | 3.76 | 3.85E-02 |  |  |  |
| hsa-miR-195-5p | 3.49 | 3.79E-02 |  |  |  |
| hsa-let-7e-5p | 3.38 | 3.45E-03 |  |  |  |
| hsa-miR-379-5p | 3.12 | 4.25E-02 |  |  |  |
| hsa-miR-125b-5p | 2.93 | 2.35E-02 |  |  |  |
| hsa-miR-193a-5p | 2.76 | 2.68E-02 |  |  |  |
| hsa-miR-98 | 2.72 | 1.76E-02 |  |  |  |
| hsa-miR-548aa | 2.36 | 3.64E-02 |  |  |  |

*compared to normal fallopian tube.
